# Silicon-Mediated Modulation of Olive Leaf Phytochemistry: Genotype-Specific and Stress-Dependent Responses

**DOI:** 10.3390/plants14091282

**Published:** 2025-04-23

**Authors:** Marin Cukrov, Velemir Ninkovic, Luna Maslov Bandić, Šime Marcelić, Igor Palčić, Mario Franić, Paula Žurga, Valerija Majetić Germek, Igor Lukić, Darija Lemić, Igor Pasković

**Affiliations:** 1 Department of Agriculture and Nutrition, Institute of Agriculture and Tourism, K. Huguesa 8, 52440 Poreč, Croatia; mcukrov@iptpo.hr (M.C.); palcic@iptpo.hr (I.P.); mario@iptpo.hr (M.F.); igor@iptpo.hr (I.L.); paskovic@iptpo.hr (I.P.); 2 Department of Ecology, Swedish University of Agricultural Sciences, SE-75007 Uppsala, Sweden; 3 Department of Chemistry, Faculty of Agriculture, University of Zagreb, Svetošimunska 25, 10000 Zagreb, Croatia; lmaslov@agr.hr; 4 Department for Ecology, Agronomy and Aquaculture, University of Zadar, Trg Kneza Višeslava 9, 23000 Zadar, Croatia; simemarcelic@unizd.hr; 5 Teaching Institute of Public Health Primorsko-Goranska County, Krešimirova 52a, 51000 Rijeka, Croatia; paula.zurga@zzjzpgz.hr; 6 Department of Food Technology and Control, Faculty of Medicine, University of Rijeka, Brace Branchetta 20, 51000 Rijeka, Croatia; valerija.majetic@medri.uniri.hr; 7 Department of Agricultural Zoology, Faculty of Agriculture, University of Zagreb, Svetošimunska 25, 10000 Zagreb, Croatia; dlemic@agr.hr

**Keywords:** foliar-applied silicon, secondary metabolism, phenolic compounds, secoiridoids, oleuropein, physiological indices, silicon utilization, effect persistence

## Abstract

Secondary metabolites in olive (*Olea europaea* L.) leaves constitute a complex framework wherein phenylpropanoids, terpenoids, and secoiridoids in particular, serve as major contributors to olive plant resilience. Silicon (Si) stands as a mediator of defense mechanisms in plants, enhancing their protective responses and adaptability. A field trial on one-year-old plantlets of two metabolically distinct olive genotypes was conducted to investigate the effects of foliar-applied Si on the phytochemical profiles of locally treated leaves. Silicon’s systemic effects in juvenile leaves were also appraised. We accounted for intervarietal differences in nutrient uptake and conducted in situ measurements of physiological indices. The peak of the summer season and the onset of autumn were chosen as the two sampling time points. Intense summer conditions prompted metabolic adjustments that resulted in phytochemical profiles unique to each cultivar. These profiles were further significantly altered by Si while remaining genotype-specific, with substantial increases in prominent compounds like oleuropein (105% and 252%) and verbascoside (62% and 126%), depending on the genotype. As the pressure from environmental factors eased, the differences in Si-mediated phytochemical responses emerged. Silicon had a limited effect on the phytochemical profile of the resilient cultivar which acquired a metabolic steady-state, while it significantly altered the profile of its metabolically more versatile counterpart, resulting with a progressive increase in its oleuropein (37%) and verbascoside (26%) levels. These effects extended to untreated, juvenile leaves as well. While effective in altering and improving the phytochemical composition of olive leaves, Si acted in a manner that adhered to each genotype’s metabolic foundation. The intensity of environmental constraints, along with each cultivar’s inherent sensitivity to them, seems to be tied to silicon’s capacity to mediate significant phytochemical alterations. The extent of silicon’s prophylactic function may therefore be dependent on a genotype’s metabolic foundation and overall sensitivity, and as such it seems inseparable from stress and its intensity.

## 1. Introduction

Olive (*Olea europaea* L.) is one of the most emblematic species of the Mediterranean basin, renowned for possessing an abundance of secondary metabolites in its leaves [1]. These compounds belong to large classes of specialized phytochemicals such as various phenolics, flavonoids, terpenoids, and notably for olive, secoiridoids. Serving as either integral or supportive molecules, these metabolites play important roles in maintaining plant homeostasis, enhancing stress tolerance, and strengthening defense mechanisms [2]. Their levels in olive leaves can vary substantially, reflecting their adaptive functions and the extent to which olive plants rely on them under changing conditions. In this sense, numerous studies have linked higher leaf total phenolic content with enhanced resilience of olive plants as there is a direct relationship between the levels of these compounds and the mitigation of oxidative damage caused by various types of stress [3,4]. Among simple phenolic compounds, tyrosol and hydroxytyrosol are recognized for their contribution to oxidative stability [5]. Phenolic acids, such as caffeic and ferulic acid, as well as verbascoside also contribute to free radical scavenging [6]. Antioxidant defenses are strongly bolstered by flavonoids as well, and many of them were shown to mitigate the adverse effects of high sunlight and UV radiation, with the highest levels typically observed during summer [7,8,9]. Olive leaves also contain a wide variety of terpenoids, with triterpenic acids, such as oleanolic acid, being the most abundant [10]. However, none of these compounds are as prominent in leaves of the olive plantlets and trees, nor as widely associated with stress relief, as the secoiridoid oleuropein. The biosynthesis of this crucial metabolite is instigated in response to various environmental stressors, including salinity, drought, heat, irradiance, or their combined occurrence [11,12,13]. Beyond its own protective roles, the conversion of oleuropein results with a range of metabolites, including other secoiridoids and simple phenolics, thus underscoring the importance of its accumulation [14]. However, the protective efficacy of all these compounds is not solely determined by their individual concentrations, but by their dynamic interactions as well [15]. Studying their responses within the context of the entire phytochemical composition is therefore more effective for understanding their functions; individual compound responses, though insightful, represent merely a part of the broader metabolic framework.

Natural growing conditions, especially in the summer season, always impose some degree of stress to which olive plants, through their phytochemical responses, constantly adapt. These responses aggregate to form phytochemical profiles, which are influenced not only by external factors but also by the olive plant’s genetic makeup and the way in which each genotype interacts with its surroundings [16,17]. Olive plants are known to exhibit great intraspecific variability in leaf phytochemical profiles, a variability that often becomes more pronounced under challenging conditions [18,19,20]. These profiles often reveal genotype-specific phytochemical patterns that can be indicative of each genotype’s metabolic plasticity [21]. In this sense, and in relation to stress, olive cultivars can differ in the inducibility of phytochemical responses or the continuation of their accumulation as some have evolved to be more dynamic in their metabolic adjustments while others maintain a more stable state [22]. These differences are mainly evolutionary consequences of historic growing conditions and selection pressures, which have, along with other traits, shaped each genotype’s metabolic versatility. As a result, olive genotypes possess varying stress tolerance thresholds [23] and may exhibit varying magnitudes of phytochemical responses when subjected to the same intensity of stress, which can be indicative of each genotype’s inherent sensitivity. This interplay of inherent traits and environmental constraints needs to be considered when evaluating the influence of some other inputs for their potential to enhance phytochemical defenses. One input that can be utilized to enhance olive leaf phytochemical adaptations and be integrated within the broad framework of the ecophysiological roles of these compounds is silicon.

Recent studies are increasingly uncovering how silicon (Si) influences the secondary metabolism of various plant species [24,25,26], yet almost none have covered how it affects the phytochemical profiles of olive leaves. Silicon is renowned for its broad structural and physiological properties that synergistically enhance plant resilience, particularly under challenging environments. Since secondary metabolites serve a protective role, some of them often align functionally with Si-mediated defense responses to form a cohesive and integrated protective framework [27,28]. On a broader scale, Si is known to potentiate the secondary metabolic pathways under various adverse conditions [25,26,28]. In some cases, Si even appears to partially substitute the protective functions of certain defensive or structural phenolic compounds [29,30]. It was shown that Si can affect the phenylpropanoid pathway and enhance those phenolic compounds that alleviate adverse effects of both abiotic [31] and biotic stress [32,33]. Moreover, Si appears to play a role in the phytohormonal regulatory network of defense-related metabolic pathways as well [34]. In the context of its ability to influence such pathways, several previous studies have shown silicon’s apparent capacity to regulate them at the transcriptional level [35,36,37]. While such effects are well-documented, these and the vast majority of other studies examining silicon’s influence on plant secondary metabolism have all been conducted under and in relation to stress. In the absence of stress, silicon’s impact on plant transcriptome is limited and its effects on plant metabolism remain latent, rarely causing significant changes [38,39]. This indicates that silicon’s primary mode of action is largely associated with a prevention of the transcriptional deregulation inherent to the stress itself rather than with its direct influence on gene expression [39]. Indeed, the majority of silicon’s effects are more likely rooted in some broad, indirect mechanism rather than in its direct molecular interference at the level of biosynthetic pathways. Therefore, instead of being an active participant in biochemical modulations, Si likely affects plant secondary metabolism through a cascading mechanism, potentially stemming from, but not limited to, its broader structural and physiological functions [39,40].

Silicon supplementation in olive trees has only recently garnered scientific attention, with foliar application emerging as an efficient way of increasing Si levels in leaves of a species typically characterized by its low accumulation [41]. Under various conditions, foliar-applied Si in olive has been shown to mitigate oxidative damage, strengthen membrane stability, increase stomatal size and density, enhance leaf water status, and support the uptake and translocation of potassium and nitrogen [42,43,44,45]. However, silicon’s effects on olive leaf phytochemical composition remain largely unexplored, particularly in open-field settings and under natural conditions where its application might hold the greatest practical relevance. Moreover, as such conditions always imply some form of stress and given silicon’s close association with it, it is unclear whether its impact on phytochemical composition is consistent across olive cultivars or varies in a genotype-dependent manner. Specifically, it is unclear whether the extent of silicon’s effects is determined by genotypic differences in metabolic plasticity and overall sensitivity to environmental conditions. To address this, we examined one-year-old olive plantlets of two metabolically distinct cultivars with known differences in the plasticity of their phytochemical responses. Also, as it is unknown if silicon’s effects in this matter are only immediate and transient or, indeed, long-lasting, we investigated them at two different time points. The peak of the summer season, when abiotic pressures are greatest, was chosen as the first leaf sampling time point, while the second was set at the beginning of autumn when these pressures eased. In this way, silicon’s effects were examined 15 and 90 days after its final foliar application.

At the later time point, we also examined if silicon’s effects on phytochemical composition of fully developed, locally treated leaves extend to juvenile, untreated leaves. Investigating these aspects in a natural setting improves our understanding of silicon’s influence on the phytochemical responses of olive plants in real-world conditions. Given its role in the enhancement of adaptive and protective capacities of plants in general, this adds to silicon’s practical relevance for sustainable olive cultivation.

The objective of this study was to (i) evaluate the effects of foliar Si application on olive leaf phytochemicals from different metabolic pathways, (ii) assess the inter-cultivar variations in metabolic responses under Si, (iii) compare the short-term (15 days) and long-term (90 days) effects of Si on the concentrations of selected compounds in locally treated, fully developed leaves, (iv) account for intervarietal differences in nutrient uptake and address the physiological state of each cultivar under open-field conditions with measurements of associated leaf spectral reflectance indices, and (v) appraise potential systemic effects by examining the phytochemical profiles and nutrient status of untreated juvenile leaves, developed from plantlets previously exposed to Si.

## 2. Results

### 2.1. Local Effects of Foliar Silicon Application

#### 2.1.1. Phytochemical Responses in Locally Treated Leaves Throughout the Experiment

**Figure 1:**
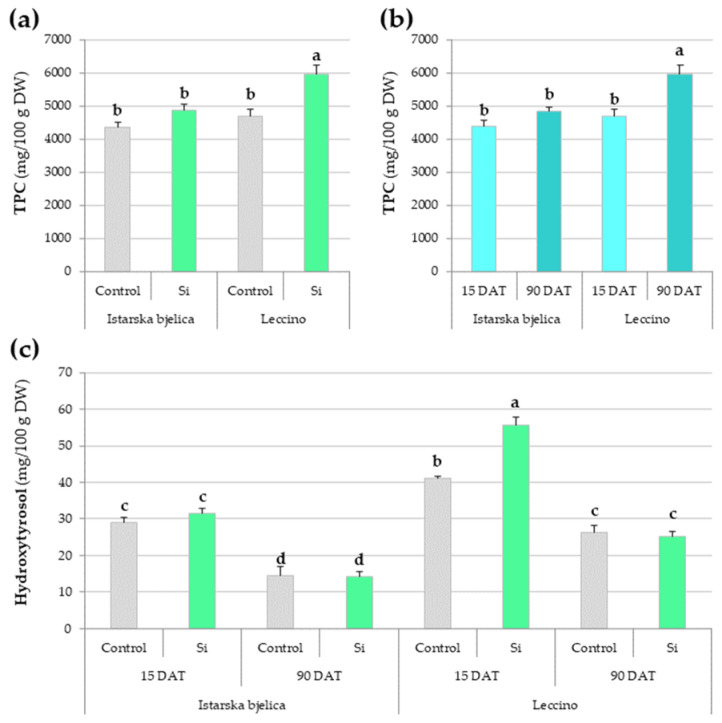
Significant interactions of: (**a**) cultivar × treatment, (**b**) cultivar × sampling time on total phenolic content (TPC), and (**c**) the effects of cultivar × treatment × sampling time on the concentration of hydroxytyrosol in locally treated leaves of two olive cultivars. Different letters above the bars represent statistically significant differences between mean values at *p* < 0.05 obtained by a two- and three-way ANOVA and Tukey’s test. DW—dry weight, DAT—days after treatment.

Significant differences in total phenolic content (TPC) were recorded for all three factors individually, namely cultivar, treatment, and sampling time (Table S1). However, their interaction was insignificant. The cultivar × treatment interaction revealed Leccino cultivar benefiting from Si application, having significantly greater TPC (5961.14 mg/100 g of dry weight (DW)) compared to Istarska bjelica (4873.7 mg/100 g DW) while their respective controls remained comparable (Figure 1a). Also, the cultivar x sampling time interaction revealed that with the passage of time, only Leccino cultivar showed a significant increase in TPC (Figure 1b).

Simple phenolic alcohols displayed compound-specific responses to the Si treatment. Hydroxytyrosol levels were influenced by all three factors (Table S1), and 15 days after treatment (DAT) Leccino showed increased levels under Si while Istarska bjelica did not (Figure 1c). Later, the treatment differences faded, although Leccino maintained higher overall concentrations. Tyrosol showed simpler alterations, with decreased levels observed both under Si treatment and over time, regardless of cultivar (Table S1).

**Figure 2:**
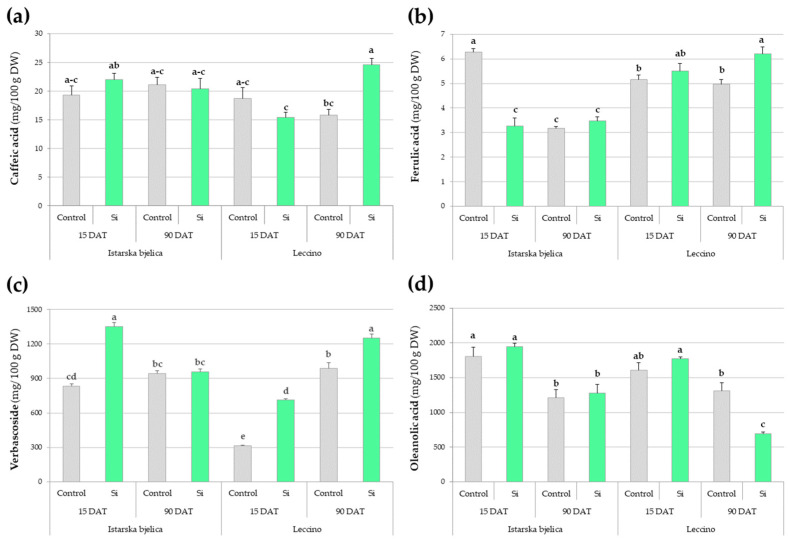
Multiple comparisons of the effects of cultivar, treatment, and sampling time combinations (highest order interactions) on the concentrations of the following: (**a**) caffeic acid, (**b**) ferulic acid, (**c**) verbascoside, and (**d**) oleanolic acid in locally treated leaves of two olive cultivars. Different letters above the bars represent statistically significant differences between mean values at *p* < 0.05 obtained by a three-way ANOVA and Tukey’s test. DW—dry weight, DAT—days after treatment.

Phenolic acids exhibited distinct cultivar-specific and temporal responses to Si treatment (Table S1). Caffeic and ferulic acids, as well as verbascoside, were influenced by all three investigated factors (Figure 2a–c). While caffeic acid levels in Istarska bjelica remained unaffected by Si throughout the experiment, leaves of the Leccino cultivar showed increased levels at 90 DAT (Figure 2a). Ferulic acid levels revealed silicon’s hindering effect in Istarska bjelica at 15 DAT, but a stimulative one in Leccino at 90 DAT (Figure 2b).

Verbascoside responses to Si treatment varied across all conditions (Figure 2c). Early on, cultivars differed in their baseline levels of verbascoside, with Istarska bjelica showing 834.57 mg/100 g DW and Leccino 313.74 mg/100 g DW, but they both displayed a substantial increase of 62% and 126% under Si, respectively. Over time, their responses diverged as the initial increase in Istarska bjelica returned to baseline while Leccino exhibited a continued accumulation in both control and Si-treated leaves, ultimately showing the highest levels (1250.25 mg/100 DW) under Si (Figure 2c). Baseline levels of oleanolic acid, a prominent triterpenic acid in olive leaves, decreased over time naturally in both cultivars. In Istarska bjelica this temporal decline was neither exacerbated nor alleviated by Si. Contrastingly, Si accentuated this decline in Leccino, resulting in the lowest levels across all conditions (Figure 2d).

**Figure 3:**
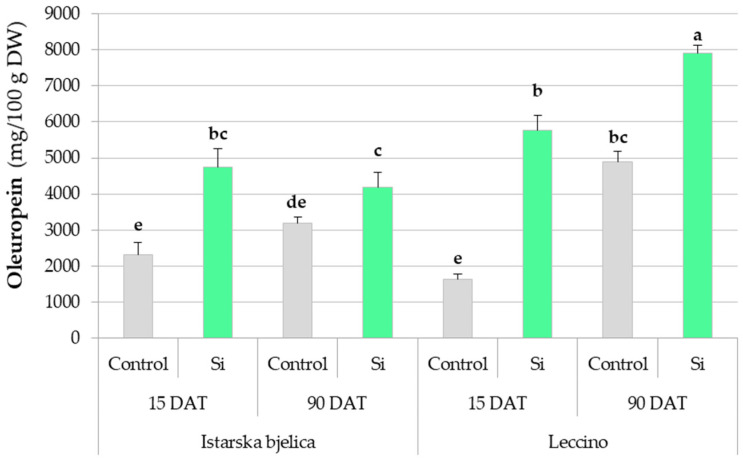
Oleuropein concentrations in locally treated leaves of two olive cultivars (Istarska bjelica and Leccino) at 15 and 90 days after treatment (DAT) in response to foliar application of silicon (Si). Shown are mean values ± standard errors. Different letters above the bars represent statistically significant differences between mean values at *p* < 0.05 according to Tukey’s test. DW—dry weight.

Oleuropein concentrations were significantly influenced by all three factors investigated (Table S2, Figure 3). Baseline levels of both cultivars increased naturally over time. Initially, the two cultivars exhibited comparable baseline levels, but as time progressed Leccino showed a greater natural capacity for oleuropein synthesis and accumulation (Figure 3). Notably, the natural increases in the oleuropein levels were modest compared to the substantial gains observed with Si application. Silicon enhanced the oleuropein levels of both cultivars early on, but later Leccino showed a markedly stronger response than Istarska bjelica. In Leccino, Si did not only sustain its already enhanced oleuropein levels recorded at 15 DAT (5766 mg/100 g DW) but facilitated a further significantly higher increase by 90 DAT (7894 mg/100 g DW (Figure 3). This was not observed in Istarska bjelica as Si merely maintained its previously recorded oleuropein levels, which were still higher compared to baseline.

**Figure 4:**
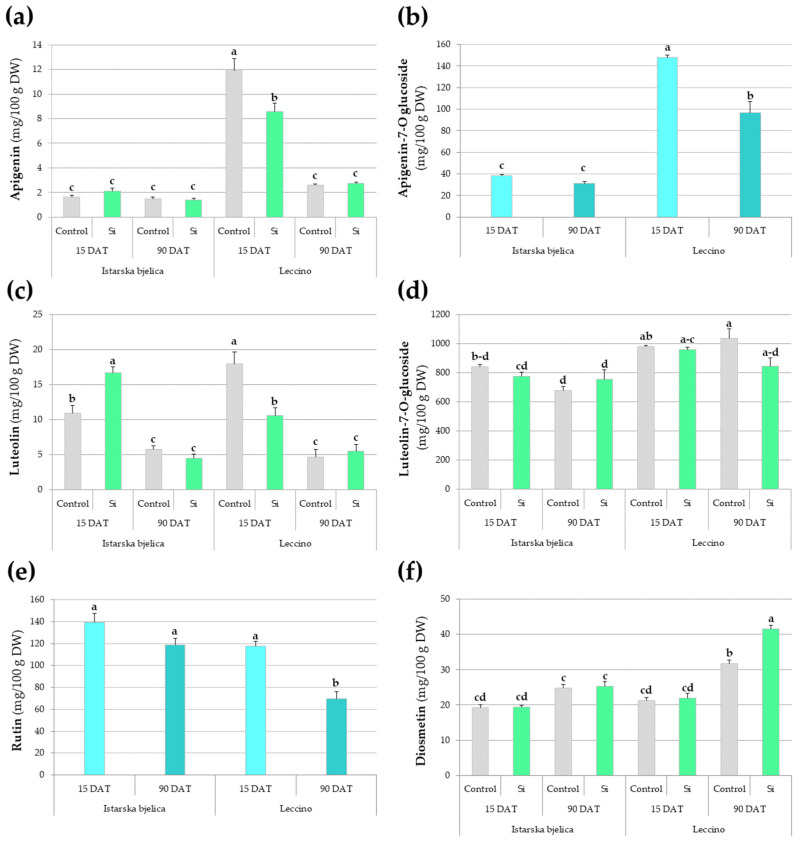
Multiple comparisons of the effects of cultivar, treatment, and sampling time combinations on the concentrations of: (**a**) apigenin, (**b**) apigenin-7-O-glucoside, (**c**) luteolin, (**d**) luteolin-7-O-glucoside, (**e**) rutin, and (**f**) diosmetin in locally treated leaves of two olive cultivars. Different letters above the bars represent statistically significant differences between mean values at *p* < 0.05 obtained by a two-way or three-way ANOVA and Tukey’s test. DW—dry weight.

The main effects of the analyzed flavonoids (apigenin and its glucoside; luteolin and its glucoside, rutin, and diosmetin) are presented in Supplementary Table S2, while their significant interactions are illustrated in Figure 4. Istarska bjelica maintained consistently low levels of both apigenin and its glucoside, while Leccino exhibited more dynamic responses (Figure 4a,b). Its apigenin levels peaked at 15 DAT in controls but decreased under Si treatment. By 90 DAT, the Leccino cultivar’s apigenin levels decreased in both control and Si treatment, aligning with the levels recorded in Istarska bjelica at the same time (Figure 4a). Additionally, apigenin’s glycosylated form showed no change under Si but maintained higher concentrations in Leccino throughout the experiment (Figure 4b).

At 15 DAT, luteolin levels increased under Si in Istarska bjelica but decreased in Leccino, with both cultivars showing a decline by 90 DAT independent of Si (Figure 4c). Luteolin-7-O-glucoside was unaffected by Si, although Leccino maintained relatively higher levels than Istarska bjelica, particularly at 90 DAT (Figure 4d). Rutin levels were also unaffected by Si, with Leccino showing a decline over time. Diosmetin levels were equally unaffected by Si early on and were comparable among cultivars. Later, they were higher in Leccino and Si furthered an increase in its levels.

#### 2.1.2. Principal Component Analysis (PCA)

**Figure 5:**
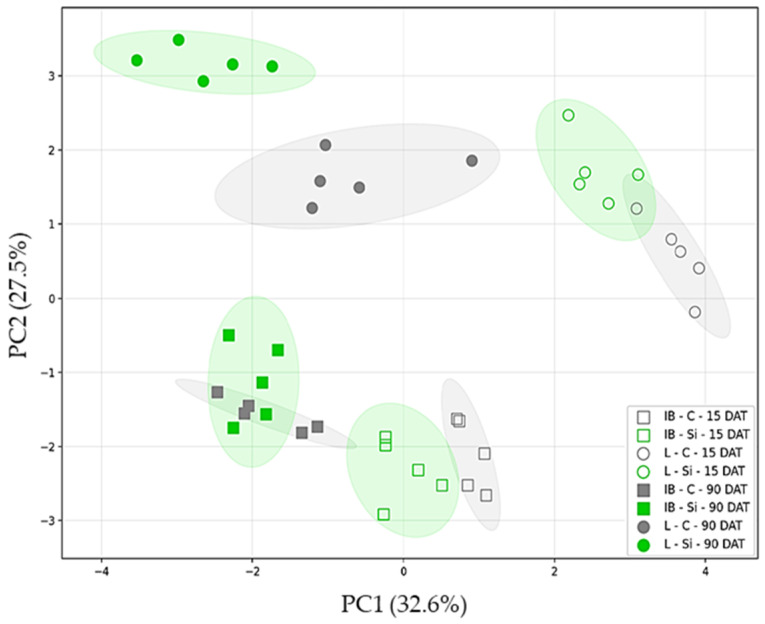
Principal component analysis of various phytochemicals in two different olive cultivars, Istarska bjelica (IB) and Leccino (L), under control (C) and silicon (Si) treatments across time (15 and 90 DAT). The score plot shows sample distribution along the first two principal components (PC1 and PC2). Markers designate cultivars (IB: squares, L: circles), colors designate treatments (C: gray, Si: green), fill designates sampling time (empty: 15 DAT, filled: 90 DAT). Ellipses represent 95% confidence intervals. Loadings table available in the Supplementary File (Table S3).

Principal component analysis (PCA) was performed on a dataset containing all of the investigated phytochemicals measured across cultivars, treatments, and sampling times. The first two principal components (PC1 and PC2) accounted for a substantial portion (60.1%) of the total variance, with PC1 and PC2 contributing 32.6% and 27.5%, respectively. Distinct metabolic differences emerged between the two cultivars, showing their clear separation across the PCA space (Figure 5). Cultivar differentiation patterns across the score plot revealed that Istarska bjelica displayed a more clustered distribution along the PC1 than its Leccino counterpart. In contrast, Leccino cultivar exhibited a greater spread along the PC1 and a higher magnitude of variation along PC2 (Figure 5). This greater spread in both dimensions indicates that the phytochemical profiles of Leccino were more variable than those of Istarska bjelica, which were more consistent.

Each cultivar showed a clear treatment-based separation at 15 DAT, albeit with minor and negligible overlaps of their respective confidence ellipses. A significant disparity in response to Si became evident between the two cultivars at 90 DAT (Figure 5). At the stated time point, Istarska bjelica demonstrated a marked overlap of its treatment groups. In contrast, Leccino exhibited a discernible response to Si, one that was consistent with its earlier pattern and marked with larger Euclidean distances later, indicating a greater separation between control and Si treatment over time.

Considered individually, both cultivars exhibited a clear temporal separation across the score plot, albeit with cultivar-specific magnitudes of differentiation (Figure 5). At both time points, Istarska bjelica maintained negative PC2 scores while Leccino displayed consistent positive scores. Furthermore, both cultivars showed a negative PC1 shift at the subsequent sampling time, yet from disparate starting points. Over time, Istarska bjelica exhibited a modest PC1 shift while Leccino displayed a more dramatic one.

#### 2.1.3. Partial Least Squares Discriminant Analyses (PLS-DAs)

**Figure 6:**
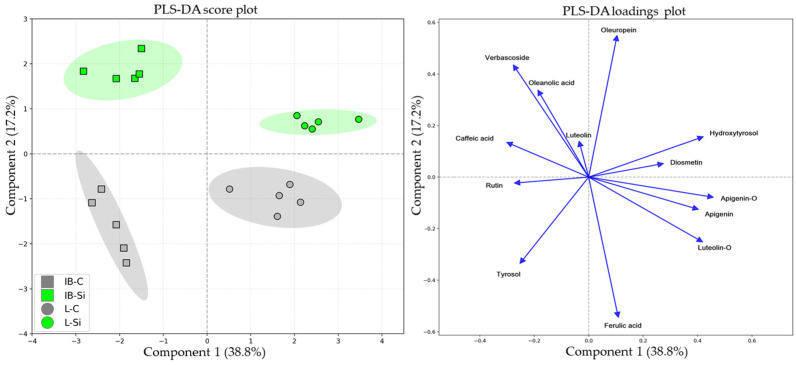
Partial least squares discriminant analysis (PLS-DA) of various secondary metabolites in the leaves of two different olive cultivars—Istarska bjelica (IB) and Leccino (L)—under control (C) and silicon (Si) treatment, sampled at 15 DAT. The score plot (**left**) shows sample distribution along the first two PLS-DA components. Ellipses represent 95% confidence intervals. The loadings plot (**right**) illustrates the relationships between the compounds and the first two PLS-DA components.

Due to the temporal differences in metabolic responses of cultivars, two separate partial least squares discriminant analyses (PLS-DAs) were conducted, one for each sampling period. This was performed to discern the compounds most responsible for group separation at each sampling time, respectively. The first PLS-DAs, performed on the data obtained at 15 DAT, revealed distinct separation patterns between both cultivars and treatments as evidenced by the score plot (Figure 6). The first component (PLSD1) accounted for 38.8%, and the second (PLSD2) accounted for 17.2% of the total variance (56.0%). The two cultivars were clearly separated along PLSD1, with genetic background emerging as the predominant factor of differentiation (Figure 6). The analysis revealed that the flavonoids apigenin, apigenin-7-O-glucoside, and luteolin-7-O-glucoside, together with hydroxytyrosol, were the most significant contributors to cultivar separation along the positive side of PLSD1 (Figure 6). Conversely, caffeic acid and rutin exhibited opposite negative loadings, indicating their role in cultivar differentiation.

The differentiation along PLSD2 was primarily driven by the metabolic responses of the cultivars to treatments. Although exhibiting divergent responses, oleuropein and ferulic acid had the highest loadings on PLSD2, thus contributing to treatment distinction the most (Figure 6). Verbascoside and oleanolic acid also contributed to PLSD2, with a similar direction of their vectors which complements the coordinated metabolic response to Si.

**Figure 7:**
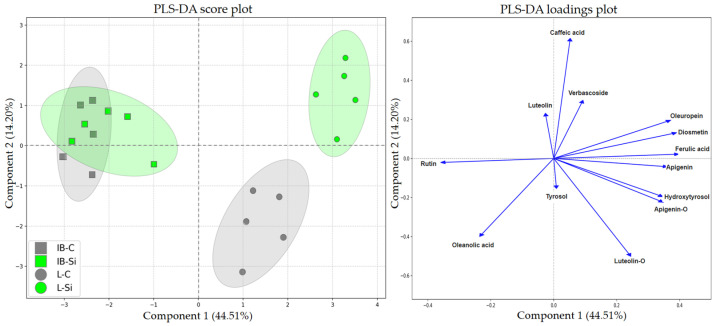
Partial least squares discriminant analysis (PLS-DA) of various secondary metabolites in leaves of two different olive cultivars—Istarska bjelica (IB) and Leccino (L)—under control (C) and silicon (Si) treatment, sampled at 90 DAT. The score plot (**left**) shows sample distribution along the first two PLS-DA components. Ellipses represent 95% confidence intervals. The loadings plot (**right**) illustrates the relationships between the compounds and the first two PLS-DA components.

A subsequent PLS-DA was performed on the data recorded at 90 DAT. The first two components captured 58.71% of the total variability, with PLSD1 accounting for the majority (44.51%) of the variation. Similar to previous analysis, the two cultivars were clearly distinguishable along the first component while additional compounds, such as oleuropein, diosmetin, and ferulic acid, defined the differentiation of cultivars. All of them were found to be directionally clustered on the positive side of PLSD1, aligning with Leccino (Figure 7). A pivotal finding emerged along PLSD2, representing a crucial aspect of this analysis. The distinct group clustering pattern showed a stark contrast between the cultivars long-term responses to Si. Istarska bjelica, which initially showed a stronger response, now demonstrated a complete insensitivity to Si as revealed by the extensive overlap of its treatment groups. In contrast, Leccino exhibited a clear treatment group separation, demonstrating a fundamentally different long-term Si response as evidenced by major shifts along both components (Figure 7).

**Figure 8:**
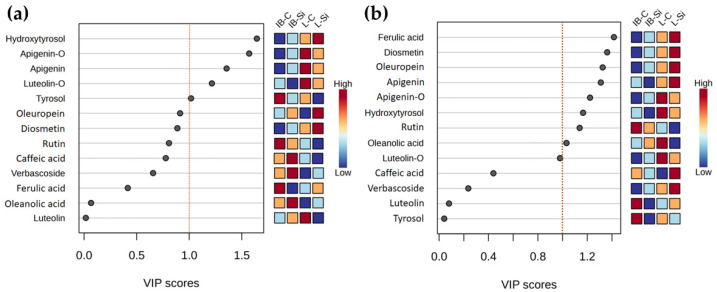
Compounds with the highest variable importance in projection (VIP) scores for the overall group separation at: (**a**) 15 DAT and (**b**) 90 DAT. The colored boxes on the right indicate the relative concentrations of the corresponding metabolite in each group under study.

The variable importance in projection (VIP) scores identified hydroxytyrosol as the primary driver of group separation along the axes of the first PLS-DAs, conducted on the data obtained at 15 DAT (Figure 8a). Subsequent to this, at 90 DAT the ferulic acid positioned itself with the highest VIP score, followed by diosmetin and oleuropein (Figure 8b). A notable observation is that at 15 DAT only 5 compounds exceeded the VIP importance threshold, while at 90 DAT this number increased to 9 compounds (Figure 8a,b).

#### 2.1.4. Silicon and Mineral Nutrients Content in Locally Treated Leaves Throughout the Experiment

The two olive cultivars did not differ significantly in their leaf Si contents (Table 1). However, they differed in their natural uptake of several macro- and micronutrients. Istarska bjelica showed a higher level of boron (B) in its leaves, while Leccino exhibited higher levels of nitrogen (N), calcium (Ca), magnesium (Mg), sulfur (S), and zinc (Zn).

Local effects of foliar Si application were evident (Table 1). As expected, a significant difference between the control and Si-treated leaves emerged. Considering this main effect, the observed variance in leaf Si content was found to be consistent with the applied treatment, with Si levels being higher in Si-treated leaves (166.88 mg/kg DW) than in control plants (107.55 mg/kg DW). Notably, the treatment x sampling time interaction revealed significantly higher Si levels in Si-treated leaves at 15 DAT (181.73 mg/kg DW) than at 90 DAT (152.03 mg/kg DW), indicating that Si levels in locally treated leaves declined over time (Figure S1a). The contents of all other elements did not differ among treatments, except for B which exhibited a significant decrease in Si-treated leaves (Table 1). The cultivar × treatment interaction revealed that Si treatment reduced the uptake of B exclusively in Istarska bjelica (Figure S1b). Even so, this cultivar maintained higher B levels than its Leccino counterpart. Over time, probably due to their upward translocation, the levels of most macronutrients (N, P, K, Mg, and S) decreased, while Ca accumulated (Table 1). Among macronutrients, significant interactions were observed only for S and the genetic background had a stronger influence on its content than treatment or sampling time, with Leccino exhibiting higher S content (Figure S1c).

#### 2.1.5. Spectral Reflectance Indices of Locally Treated Leaves Throughout the Experiment

**Figure 9:**
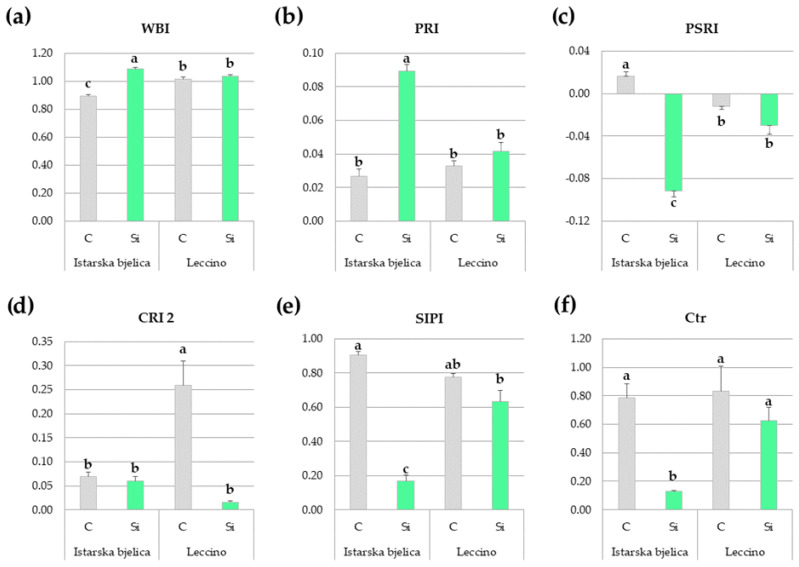
Multiple comparisons of the initial effects of cultivar and treatment combinations on leaf spectral reflectance indices of two olive cultivars, locally treated with control (C) and silicon (Si) treatments. Indices include: (**a**) water band index—WBI, (**b**) photochemical reflectance index—PRI, (**c**) plant senescence reflectance index—PSRI, (**d**) carotenoid reflectance index 2—CRI 2, (**e**) structure insensitive pigment index—SIPI, (**f**) Carter index—Ctr. Different letters above the bars represent statistically significant differences between mean values at *p* < 0.05 obtained by a two-way ANOVA and Tukey’s test.

Prior to each sampling, several leaf spectral reflectance indices were measured with a portable spectrometer for an in situ evaluation of plant physiological parameters and overall status. These measurements also provide insights into potential stress which would have been experienced uniformly by all plants given the open-field setting of the experiment. Twelve reflectance parameters were measured across different categories, with indices related to water content (WBI), light use efficiency (PRI), senescent carbon (PSRI), carotenoids (CRI 1, CRI 2), chlorophyll concentration (NDVI, CNDVI), and several other associated and stress-related indices (SIPI, Ctr, NPQI, VREI, and ZMI). Data obtained 15 days after the onset of the experiment, at the first sampling time (Table S4), exhibited markedly higher variability compared to the data obtained at the second sampling time (Table S5). At 15 DAT, significant cultivar × treatment interactions were observed for half of the measured indices, and these are presented in Figure 9.

Istarska bjelica seemed to have benefited from Si application more than the Leccino cultivar as it exhibited a significantly higher water band index (WBI), photochemical reflectance (PRI), and reduced senescence (PSRI) in Si-treated leaves compared to the controls (Figure 9a–c). The control leaves of the Leccino cultivar displayed the highest carotenoid reflectance (CRI 2), but Si treatment effectively mitigated this (Figure 9d). The structure insensitive pigment index (SIPI) serves as a good indicator of stress as it measures the ratio of carotenoids to chlorophyll in leaves, providing insights into the physiological status of the plant. The two cultivars exhibited similar baseline SIPI levels, indicating greater stress experienced by the controls than the Si-treated leaves. However, silicon’s stress-mitigating effect was more pronounced in Istarska bjelica than in Leccino (Figure 9e). The Carter index (Ctr) values, which increase as chlorophyll degrades, paralleled SIPI trends (Figure 9f). In addition, Si-treated plants showed lower values of normalized pheophytinization index (NPQI), thus indicating less chlorophyll degradation compared to controls with no significant differences among the cultivars (Table S4).

Contrastingly to data recorded at 15 DAT, no significant interactions were observed at 90 DAT nor were there any statistically significant differences between the treatments in any of the measured indices (Table S5). However, significant differences between cultivars emerged, showing Leccino with lower PRI and higher SIPI and Ctr indices, indicating that its physiological state was hindered by the open-field conditions slightly more than that of Istarska bjelica (Table S5).

### 2.2. Systemic Effects of Foliar Silicon Application

#### 2.2.1. Phytochemical Responses of Juvenile, Untreated Leaves

Following the second sampling time point, metabolites analyzed in directly treated (local) leaves were also investigated in juvenile, untreated leaves that had developed from plantlets previously exposed to either a control or Si treatment. This was performed to evaluate potential systemic impact of Si application. Juvenile leaves exhibited no significant differences in TPC between cultivars or treatments (Table 2). Among cultivars, Leccino exhibited higher levels of hydroxytyrosol, caffeic acid, glycosylated flavonoids, and diosmetin, while oleanolic acid and rutin were more abundant in Istarska bjelica.

Silicon’s systemic effects were most evident in significantly higher levels of verbascoside and oleuropein (Table 2). Though its concentration did not differ between cultivars, juvenile leaves which developed from Si-treated plantlets exhibited nearly twofold higher levels of oleuropein compared to those of the control group. This outcome closely mirrored the findings in locally treated leaves, indicating a clear similarity in silicon’s influence over the most prominent secoiridoid and a profound systemic effect.

Flavonoids apigenin and luteolin showed a substantial decrease in juvenile leaves of Si-treated plantlets while their glycosylated forms showed no significant differences among treatments. Furthermore, all significant two-way interactions that were observed among flavonoids (Table 2) showed a predominant influence of genetic background over treatments.

#### 2.2.2. Silicon and Mineral Nutrients Content in Juvenile, Untreated Leaves

Si content in juvenile leaves showed no differences either among cultivars or treatments (Table 3). Its levels were nearly half of those recorded in fully developed local leaves. The juvenile leaves that developed from the Leccino cultivar exhibited higher levels of N, P, K, Ca, Mg, S, and Zn than those of Istarska Bjelica, which only had a higher B content. Of all the nutrients analyzed in juvenile leaves, B was the only one displaying a significant difference between treatments. Specifically, juvenile leaves from Si-treated plantlets had significantly lower B levels compared to untreated plantlets (Table 3).

**Figure 10:**
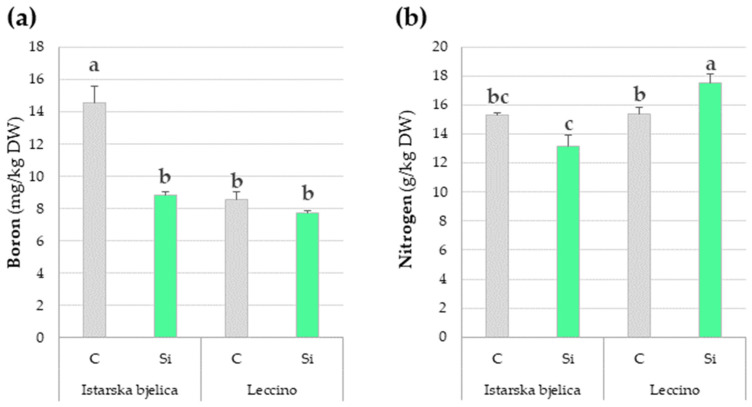
Comparisons of significant effects of cultivar and treatment combinations (two-way interactions) on contents of boron (**a**) and nitrogen (**b**) in juvenile leaves of two olive cultivars (Istarska bjelica and Leccino), previously exposed to control (C) and silicon (Si) treatments. Different letters above the bars represent statistically significant differences between mean values at *p* < 0.05 obtained by a two-way ANOVA and Tukey’s test. DW—dry weight.

However, this decrease was specific to Istarska bjelica only, as revealed by the cultivar × treatment interaction (Figure 10a). Furthermore, the analysis also revealed an important interplay between the two main factors influencing the content of N. The interaction revealed that juvenile leaves from Si-treated plantlets exhibited significantly higher N content compared to control plants, with this effect being exclusive to the Leccino cultivar (Figure 10b). To summarize, foliar application of Si hindered the translocation of B to juvenile leaves of Istarska bjelica while it enhanced the translocation of N to juvenile leaves of the Leccino cultivar (Figure 10). Therefore, though restricted to just B and N, silicon’s systemic effect on leaf mineral content was evident in juvenile, untreated leaves that developed from plantlets previously exposed to foliar Si treatments.

## 3. Discussion

### 3.1. Research Highlights

Initially, summer field conditions prompted metabolic adjustments that resulted in phytochemical profiles unique to each olive cultivar. These profiles were further significantly altered by silicon (Si) while remaining equally unique. Thus, the impact of its foliar application was evident and, along with other effects, reflected silicon’s ability to effectively modify the adaptive capacities of olive cultivars during challenging summer conditions. However, at the onset of autumn, as the pressure from environmental factors eased, the intervarietal differences in Si-mediated responses emerged. Silicon had little effect on the phytochemical profile of the more resilient and metabolically less responsive cultivar, meanwhile it significantly altered the profile of its counterpart with inherently greater metabolic plasticity. The results presented here indicate that Si, while being effective in altering and even improving the phytochemical composition of olive leaves, does so in a manner that adheres to each genotype’s metabolic foundation. Moreover, the intensity of environmental constraints, along with each cultivar’s inherent sensitivity to them seems, to be closely tied to silicon’s capacity to mediate significant phytochemical alterations. In instances where these constraints are sufficiently intense, Si may have a strong influence on diverse olive genotypes, shaping phytochemical profiles that differ from those formed without Si and are yet tailored to the distinct characteristics of each genotype. When this intensity diminishes and no longer sufficiently challenges the metabolic steady-state of more resilient cultivars, or if they acquire such a state sooner, silicon’s effects on their phytochemical profiles may be negligible or even absent. The observable effects may then be evident in more sensitive cultivars, or in those that rely more heavily on their phytochemical defenses. Alternatively, the effects may be observable in cultivars that are actively adjusting to their surroundings. In such cases, silicon’s influence on their leaf phytochemical composition can still be long-lasting and as further demonstrated they can even be systemic. These findings support the notion of silicon’s prophylactic function, which is a function that is intrinsically tied to each olive genotype’s metabolic foundation, phytochemical plasticity and overall sensitivity, and as such is seemingly inseparable from stress and its intensity.

### 3.2. Short-Term Effects of Foliar-Applied Si

The principal component analysis (PCA), performed on the data obtained throughout the experiment, marked Istarska bjelica as a metabolically less responsive cultivar with a lower phytochemical plasticity (Figure 5). In contrast, Leccino cultivar showed greater metabolic plasticity and a stronger preference for phytochemical adaptations to open-field conditions. This was in alignment with our previous studies where Istarska bjelica exhibited the lowest polyphenolic plasticity in comparison to several other olive cultivars, including Leccino [16,46]. Even so, under intense environmental pressures of the summer season, both cultivars exhibited clear short-term responsiveness to Si, showing phytochemical profiles distinct from their respective controls, despite the genetic differences in metabolic versatility (Figure 6). At the broadest analytical level, the two cultivars initially had similar total phenolic contents (Figure 1b). Simultaneously, the Si-mediated changes in quantitatively less prevalent compounds were genotype-specific. Istarska bjelica was associated with a chemically more varied array of metabolites, while Leccino was linked with hydroxytyrosol and the majority of flavonoids. The latter aligns with several other studies showing the Leccino cultivar’s higher flavonoid plasticity in relation to Istarska bjelica and some other olive cultivars [16,46,47]. The concentrations of more prominent compounds increased under Si in both cultivars significantly. In Istarska bjelica, verbascoside and oleuropein rose by 62% and 105%, respectively, while in Leccino, verbascoside increased by 126% and oleuropein by an outstanding 252% (Figure 2c and Figure 3). The accumulation of these compounds, particularly oleuropein, signifies an adaptive response commonly associated with improved abiotic stress tolerance in various olive genotypes [48,49]. Considering the summer field conditions to which the olive plantlets were exposed, the latter reinforces the significance of silicon’s influence on those compounds. In a recent study, during the summer season, the foliar application of Si in mature Leccino trees also led to a short-term and significant increase of oleuropein, yet in a considerably lower percentage [50]. This suggests that beyond environmental factors, the magnitude of the olive plant’s Si-mediated response can also be influenced by its age, as one study reported higher levels of oleuropein and phenolic compounds in younger trees compared to older ones [51]. Furthermore, while the levels of verbascoside and oleuropein increased in both cultivars, the percentage increase observed in Leccino was twice that of Istarska bjelica for both compounds. This is indicative of the Leccino cultivar’s higher biosynthetic capacity which could have been further fueled by its inherently higher uptake of some essential nutrients (N, Ca, Mg, S, and Zn, Table 1), a previously documented trait [16]. Nevertheless, the Si-mediated genotype-specific responses of less prevalent compounds, together with the varying magnitudes of response in more prominent compounds, led to distinct phytochemical profiles among investigated olive cultivars. This suggests that silicon’s efficiency in this matter can be closely associated with the genetic makeup and the metabolic foundation of each olive cultivar.

The genotypic differences with regard to Si were not confined to its phytochemical efficiency but also extended to the distinct ways in which cultivars utilized Si. Despite having similar leaf Si contents, with a 55% increase relative to untreated plants, the extent of silicon’s short-term benefits in terms of physiological indices differed markedly among cultivars. In this aspect, the more resilient cultivar seems to have channeled Si in a way which was more effective in improving its leaf water status and photosynthetic efficiency, while delaying the leaf senescence (Figure 9a–c). The key ways in which Si improves leaf water status are reflected in its ability to facilitate osmotic adjustments, reduce membrane permeability, enhance its structural integrity, increase turgidity, and thicken the cuticular layer [52,53]. In this context, though specific to Istarska bjelica which had an inferior water status to begin with, our results align with those from a recent study on olive trees [42] where foliar application of nano-Si effectively enhanced leaf relative water content. Additionally, the majority of leaf water loss occurs through the stomata, and in olive trees Si was recently shown to influence stomatal density and size [44]. In relation to this, Si can also improve leaf water use efficiency [54], gas exchange attributes [55], mesophyll conductance [56], and protect photosynthetic apparatus and pigments [57]. Such effects have been reported in various plant species, while the observed improvements in leaf photosynthetic efficiency and senescence (Figure 9b,c) suggest that Si may impart some of these benefits to olive plants as well. Still, why these effects were so prominent in only one cultivar warrants a further investigation. Moreover, as evidenced by indices related to carotenoid-to-chlorophyll ratios and chlorophyll degradation (Figure 9e,f), the Si-treated leaves of Istarska bjelica outperformed those of Leccino as well. Considering that silicon’s benefits in plant physiology are well known, improvements in these indices were anticipated. However, intervarietal differences to this extent were not expected. Still, it is well known that to protect against adverse conditions olive leaves undergo structural adaptations which manifest through changes in tissue morphology, an increase in cell density, and a decrease in cell size [58,59]. Since the degree of these adaptations varies among genotypes, the extent of silicon’s reinforcing influence may also differ accordingly. It is possible that the previously recognized higher resilience of Istarska bjelica stems from its higher proclivity for such structural adaptations, though this has yet to be confirmed. Considering silicon’s structural roles and its close association with the extracellular matrix [52], it is plausible that Si reinforces these adaptations in alignment with each cultivar’s inherent commitment to them. While requiring further confirmation, this could explain the observed genotypic differences in physiological performance under Si. Since there were no intervarietal differences in the leaf Si contents of treated plantlets, this underscores the distinct ways in which Si can be utilized by olive cultivars. However, it is important to note that these effects were transient and that no Si-mediated improvements in these traits were observed 90 days after silicon’s foliar application.

### 3.3. Long-Term Effects of Foliar-Applied Si

While the two cultivars exhibited a comparable magnitude of Si-mediated phytochemical adjustments to challenging summer conditions, their long-term responses as these pressures eased were markedly different. As evidenced by the significant overlap of its two treatment groups (Figure 7), Si had a negligible impact on the phytochemical profile of the metabolically more conservative cultivar. In contrast, its counterpart with inherently greater metabolic plasticity exhibited a sustained and distinct response, marked by clear separation between its treatment groups and a significant increase in total phenolic content under Si (Figure 1a). This suggests that Si, applied 90 days earlier, facilitated a lasting phenolic response that persisted even though the leaf Si content declined (Figure S1a). In response to abiotic stressors, Si-mediated increases in individual or total phenolics have been observed in various plant species [31,60], though such effects hardly persist this long after its application. In contrast to short-term results, the majority of compounds now showed greater variability in metabolically more versatile cultivar, underscoring its reliance on phytochemical adaptations throughout the experiment. This occurred to such an extent that nearly all analyzed metabolites proved important for group discrimination, as evidenced by their variable importance scores (Figure 8b). Importantly, verbascoside and oleuropein levels again showed the most remarkable increases with Si, reflecting the necessity for continued adaptations even as the intensity of environmental constraints eased and signifying their protective roles [48,49]. By this stage, verbascoside levels in Istarska bjelica had subsided, while the Leccino cultivar’s Si-treated leaves showed a 26% increase (Figure 2c). Strikingly, its oleuropein levels increased by 61% relative to controls which represented a further 37% rise compared to earlier results (Figure 3). This demonstrates that the Leccino cultivar’s innate phytochemical adaptability surpassed that of Istarska bjelica, with Si further amplifying this process as conditions required an increase in these compounds earlier or still required it later. Thus, the way in which each cultivar handles its surroundings may influence both its phytochemical response and the extent to which Si may act upon on it. Interestingly, silicon’s peak effects on oleuropein, aside from being genotype-specific, actually emerged much later rather than earlier (Figure 3). This delayed yet such profound influence on its biosynthesis is indicative of silicon’s indirect mode of action. If Si had a direct influence on oleuropein synthesis at the molecular level, its peak effects would likely have appeared much earlier. Silicon’s non-essentiality [61] and limited biochemical activity in terms of direct interactions with enzymes involved in biosynthetic pathways make this improbable [39,61]. Moreover, secoiridoid synthesis is regulated at the transcriptional level [62], and Si by itself has a limited influence on the plant transcriptome in the absence of stress [38,39]. On the other hand, stress itself induces extensive transcriptional changes [63]. Therefore, stress was the driving force behind these phytochemical responses while the genetic makeup and adaptability of each cultivar to its subsequent lower intensity shaped the extent of silicon’s influence on those responses. This was an influence which adhered to each genotype’s sensitivity whilst also closely depending on it. This would further support the notion that Si acted on olive phytochemical defenses through an indirect cascading effect which was, much like silicon’s general roles, invariably tied to stress.

In line with its documented lower polyphenolic plasticity [16,46], the acquired metabolic steady-state of Istarska bjelica likely had no external incentive potent enough to elicit its phytochemical counter-response which would then be mediated by Si. In contrast, the Leccino cultivar’s inherently greater reliance on phytochemical adaptations resulted in a sustained heightened metabolic state and its remarkable plasticity with respect to Si had a lasting effect on its phytochemical defenses. Similar short-term responsiveness with respect to Si, observed among cultivars earlier, can be attributed to a higher magnitude of stress encountered by all plants at the height of the summer season. This magnitude was strong enough to elicit phytochemical coping mechanisms in both cultivars, which were subsequently mediated by Si. Ultimately, the metabolic foundation seems to dictate how Si is functionally integrated into its existing framework. Variations in response inducibility are prominent among olive genotypes as some have evolved to dynamically adjust their metabolism while others maintain a more stable state [20,22]. Thus, the genetic makeup is an influential determinant of silicon’s impact on olive metabolism, but it is interrelated with the environmental context as well. In other plant species, genotype-dependent differences in silicon’s effects may stem from variations in leaf micromorphology [64], its subcellular localization [65], use efficiency, and utilization [66], but these aspects have yet to be examined in olive trees. We anticipate that future research will shed light on these aspects, enabling us to provide more comprehensive inferences about how various olive genotypes utilize Si and how that relates to their phytochemical adaptations. Still, given the scope of its overall influence demonstrated here, Si proved why it holds the laurel of a beneficial element.

### 3.4. Silicon’s Systemic Effects

At the end of the experiment, juvenile leaves that developed from plantlets previously subjected to control or Si treatment were sampled and their phytochemical profiles (Table 2) and mineral contents (Table 3) were compared. No significant differences in total phenolic content were observed among cultivars or treatments. However, verbascoside and oleuropein, which increased significantly in locally Si-treated leaves, exhibited a similar trend in the juvenile leaves of the same plantlets. The levels of oleuropein were particularly remarkable, exhibiting a two-fold increase compared to controls (Table 2). This indicates that its significantly higher accumulation in locally treated leaves of plantlets previously sprayed with Si effectively translocated to juvenile leaves. Given its roles in olive resilience [11,12,48,49], this would mean that even the newly grown foliage, long after silicon’s application, may better cope with oncoming challenges. Genotypic differences in the phytochemical composition of juvenile leaves were observed in levels of oleanolic acid and the majority of flavonoids, the latter being predominant in Leccino.

Silicon content in juvenile leaves was not different among control and Si-treated plantlets (Table 3), which is consistent with its inability to translocate to new leaves once it deposits [67]. Juvenile leaves of Leccino exhibited higher concentrations of all macronutrients (N, P, K, Ca, Mg, and S) and Zn, reflecting most of the observations in fully developed leaves and confirming intervarietal differences in mineral composition [16]. Further, a reduction of B content was initially observed in fully developed leaves of Istarska bjelica treated with Si (Figure S1b), which later extended more prominently to the juvenile leaves (Figure 10a). This indicates that Si impaired its shoot translocation, aligning with reports on other plant species where it was shown that Si immobilizes B [68]. On another note, foliar-applied Si caused an increase of N content in juvenile leaves of Leccino (Figure 10b). This observation closely aligns with a recent report on olive [45], which found that Si enhanced N uptake in a different cultivar, albeit only in conditions with limited nitrogen availability. The underlying reasons for these intervarietal differences in silicon’s effects on leaf mineral composition remain to be further investigated. Still, the observed increase in verbascoside and oleuropein, along with genotype-specific interactions with B and N, attest to silicon’s profound systemic effects.

## 4. Materials and Methods

### 4.1. Experimental Set-Up and Foliar Treatments

The experiment was set-up in an open-field on the Croatian island of Ugljan (44°07′63″/15°19′17″/70 m) as a random block design with three main factors, namely cultivars (Istarska Bjelica and Leccino), treatments (control and Si), and the sampling time (15 and 90 days after treatment (DAT)). One-year old olive plantlets from two olive cultivars (Cv.), Istarska Bjelica and Leccino, were examined. Twenty-five plantlets for each cultivar-treatment combination, split into five replicates, were planted in rows of 20 by 50 cm in soil on the 24th of March, 2020. Foliar silicon (Si) treatment consisted of water and Silitec (Kimitec Agro^®^) at a concentration of 8.5 mL per liter of water (1.1 g Si/L), according to manufacturer’s (Kimitec Agro, Vicar, Almeria, Spain) recommendations. In the obtained solution, 1 mL of a wetting agent (Tensiofill, K-Adriatica, Loreo, Italy) was added. Control treatments consisted of a regular tap water supplemented with 1 mL of the same wetting agent as well. The plantlets were sprayed until the leaves were completely covered with the solution, until run-off. Leaves that were fully developed were treated directly with either water (control) or the Si treatment, which was performed to assess the local effects of silicon’s foliar application. For the purposes of this study, these leaves were herein referred to as ‘local leaves’. Juvenile leaves that developed from plantlets whose local leaves were previously subjected to either the control or Si treatment and which were not directly exposed to either treatment are referred to as ‘systemic leaves’. The first foliar treatment was applied on the 13th of June, and the second and third were both applied at 15-day intervals from the previous one on the 28th of June and on the 13th of July. The experimental period began following the final application of foliar Si treatment on July 13th and lasted for a total of 90 days, until October 10th. Before the experimental period began, a drip irrigation system was installed to provide 1.5 L of water per plantlet daily at regular intervals.

### 4.2. Soil Properties

Soil analysis was performed at the Enological-Pedological Laboratory at the University of Zadar, Croatia. Soil samples were taken from a 0–30 cm depth, revealing the following results: soil pH of 7.8 in water and 7.3 in KCl, total carbonates at 11% CaCO_3_, active lime at 4% CaO, organic matter content of 2.3%, nitrogen (N) at 0.17%, phosphorus (P_2_O_5_) of 8 mg/100 g, and potassium (K_2_O) of 27 mg/100 g. Similarly to procedures described in a previous study [69], the aforementioned properties were determined using standardized methods: the soil reaction (pH) was measured following ISO 10390:2005, total carbonate content was assessed by the volumetric method in accordance with ISO 10693:1995, while active lime was measured using the Galet method. Organic matter was quantified using ISO 14235:1998, while the total nitrogen was determined based on ISO 11261:1995. Additionally, plant-available phosphorus and potassium were evaluated with the Egner–Riehm–Domingo method.

### 4.3. Environmental Variables

**Figure 11:**
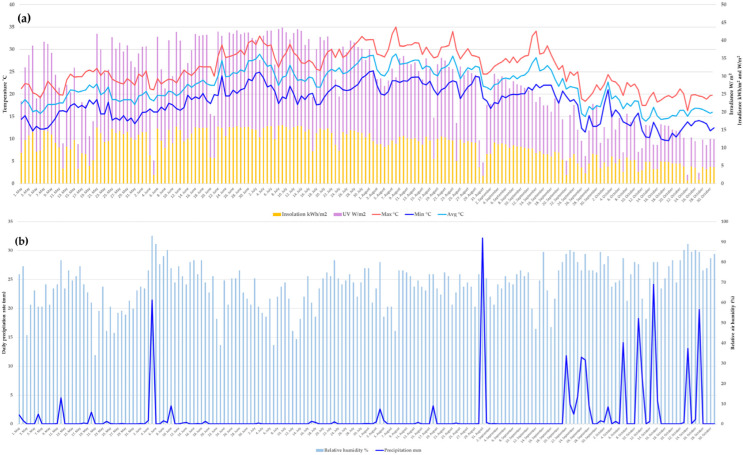
Environmental variables: (**a**) insolation (kWh/m^2^), UV (W/m^2^), maximum, minimum, and average daily temperatures (°C); and (**b**) relative air humidity (%) and precipitation during the experimental period.

Environmental parameters were continuously monitored from before the treatments began, throughout the treatment period, and for the entire duration of the experiment. (Figure 11a,b). The measured parameters included solar and UV irradiance, maximal and minimal temperature, relative air humidity, and precipitation rate. The parameters were measured by the portable weather station 3R AWS050 (Darrera S. A., Barcelona, Spain).

### 4.4. Cultivar Characterization

Istarska bjelica, an indigenous cultivar from Croatia’s Istrian region, and the allochthonous Italian cultivar Leccino were used to compare intervarietal differences to the applied treatment. Both cultivars are characterized by strong vigor, high canopy density, consistent and high productivity and favorable cold stress tolerance [16]. Leaves of the Istarska bjelica cultivar are elliptic, lanceolate with a medium width, similar to Leccino leaves, only longer. Both cultivars are partially self-incompatible, producing medium and ovoid fruits [16].

### 4.5. Leaf Spectral Reflectance Measurements

Prior to each sampling time, leaf spectral reflectance measurements were carried out by a Ci-710s Miniature Leaf Spectrometer (CID Bio-Science, Camas, WA, USA). Four fully developed leaves per plant were used for the measurement of vegetation indices (VIs) across a 350–1100 nm reflectance span, a resolution in visible and near-infrared radiation (VIS-NIR). The measurements were conducted from 0900 to 1130. The mid part of each leaf (adaxial side) was used for measurements. Three biological replicates (three different plants) with four technical replicates (four leaves per plant) per treatment were used for reflectance measurements. A total of 12 VIs were used to assess the physiological state of plantlets, which was similar to a previous study on olive by Sun et al. [70] but modified as listed with the respective formulae and references in Table 4. Reflectance spectra were processed using SpectraSnap! v. 1.1.3.149 software, provided by the manufacturer (CID Bio-Science, USA).

### 4.6. Plant Material Sampling and Preparation

In order to determine the short-term effects of silicon on foliage that have been locally treated, fully grown olive leaves from the middle section of the plantlets were sampled 15 days after the final Si treatment (15 DAT) on July 28th. In order to determine the long-term effects of silicon, other fully grown leaves were sampled at the end of the experimental period, 90 days after its final administration (90 DAT) on October 10th. These leaves also represent local leaves as they were part of locally treated, directly sprayed foliage. At 90 DAT, systemic leaves—young, untreated foliage that developed from plantlets previously subjected to either of the two treatments—were also sampled. At each sampling time (15 or 90 DAT), immediately after sampling the leaves were washed in 1% acetic acid, dissolved in deionized water of the highest purity (type I), and then double rinsed in deionized water, which was obtained from a Siemens UltraClear water purification system (Siemens AG, Munich, Germany). After rinsing, leaves were placed in paper bags and dried at 35 °C (UF-260 Universal Oven, Memmert GmbH, Büchenbach, Germany) until constant mass, consistent with a previously described protocol [16]. Dry leaves were finely milled using a centrifugal mill (Retsch Ultra Centrifugal Mill ZM 200, Düsseldorf, Germany) and stored in closed 50 mL plastic tubes in the dark at room temperature.

### 4.7. Chemicals

Methanol (MeOH) and acetonitrile (AcN) were purchased from Merck (Darmstadt, Germany) and phosphoric acid from Sigma-Aldrich (St. Louis, MO, USA). Analytical grade standards of apigenin, apigenin-7-O-glucoside, caffeic acid, diosmetin, ferulic acid, hydroxytyrosol, luteolin, luteolin-7-O-glucoside, oleanolic acid, oleuropein, rutin, tyrosol, and verbascoside, as well as the Folin–Ciocalteu reagent were procured from Sigma-Aldrich (St. Louis, MO, USA). Hydrochloric acid (Suprapure) was purchased from Merck (Darmstadt, Germany). HPLC grade deionized water was obtained from Siemens UltraClear (Siemens AG, München, Germany). The multielement standard solution was obtained from Perkin Elmer (NexION Setup Solution, Waltham, MA, USA). Argon used to form plasma for ICP-MS was supplied by Messer (Messer Croatia Plin d.o.o., Zaprešić, Croatia) and was of purity 6.0 as well as acetylene.

### 4.8. Extraction of Olive Leaf Secondary Metabolites

Extraction of olive leaf secondary metabolites was performed in accordance with a previously described procedure [16]. A finely milled sample (500 mg) was weighed (Radwag AS 310.X2, Radom, Poland) inside glass vials. Using 25 mL of methanol/water (80:20, *v*/*v* MeOH, Merck, Germany), the extracts were prepared in an ultrasonic bath (frequency 35 kHz, power 125 140/560 W, Sonorex Digitec, Bandelin electronic, Berlin, Germany) for 20 min. Extract aliquot (15 mL) was then centrifuged for 10 min at 7000 rpm (Centric 350, Domel, Železniki, Slovenia). The hydrophilic supernatant fraction was filtered through a 0.45 µm-pore cellulose acetate filter and used for further analysis.

### 4.9. Determination of Total Phenolic Content (TPC)

Determination of the total phenolic content in olive leaf extracts was based on the reaction of coloration of phenols with the Folin–Ciocalteu reagent and sodium carbonate, similar to our previous study [16]. The reaction included 250 µL of olive leaf extract with the addition of 15 mL of deionized water and 1.25 mL of Folin–Ciocalteu reagent in 25 mL flasks. The reaction mixture was neutralized by adding 2.5 mL of saturated sodium carbonate solution after 3 min. After 90 min of incubation in the dark at room temperature, the absorbance was measured at 760 nm using a UV/VIS Lambda Bio 40 spectrometer (Perkin-Elmer, Waltham, MA, USA). The concentration of total phenols was determined based on a calibration curve of pure caffeic acid (Sigma Aldrich, Steinheim, Germany), within a concentration range from 0.0256 to 1.0 mg/mL (r2 = 0.9998). All measurements were carried out in triplicate and the results were expressed as mg of caffeic acid equivalents per 100 g of dry matter.

### 4.10. Identification and Quantification of Olive Leaf Secondary Metabolites by High-Performance Liquid Chromatography (HPLC)

Targeted phenolic compounds were determined by high-performance liquid chromatography (HPLC) using a Perkin-Elmer HPLC Series 200 chromatograph (Walthamm, MA, USA) coupled with an autosampler, a binary pump, a vacuum degasser, a column oven, and a UV/VIS detector. The separation of phenols was performed using an Ultra Aqueous C18 column (5 µm, 150 × 4.60 mm, Restek, Bellefonte, PA, USA). A solvent system composed of 0.2% phosphoric acid as solvent A and a mixture of methanol and acetonitrile (1:1, *v*/*v*) as solvent B was used for separation. The chromatographic conditions were set as follows: the extract injection volume was 20 µL, the column temperature was set to 25 °C, and a flow rate of 0.8 mL/min was applied. The separation program started with 96% solvent A, followed by a decrease down to 50% after 40 min. Additional drop of solvent A by 10% was achieved after 5 min, while in a period from 45 to 60 min the solvent A proportion was down to 0. For the next 8 min, solvent B passed through the column. A reversed linear increase back to 96% was programmed from 68 to 70 min, and the attained mobile phase composition was retained for the last 10 min to achieve the stability of the column. All phenolic compounds were identified and quantified by comparing their retention times and peaks area with those of analytical, pure grade standards. Five calibration levels were made by appropriate dilutions of the stock standard solutions and calibration curves with r2 ≥ 0.999 were accepted for concentration calculation. The resultant concentrations of phenolic compounds were expressed as mg 100 g^−1^ DW. The UV/Vis detection was set at 250 nm for luteolin-7-O-glucoside and oleuropein. A wavelength of 280 nm was used to detect and quantify hydroxytyrosol, tyrosol, and apigenin-7-O-glucoside. Caffeic and ferulic acids, verbascoside, and apigenin were detected at 305 nm, while 370 nm was used for luteolin and rutin. Oleanolic acid was detected at 210 nm and diosmetin at 350 nm.

### 4.11. Elemental Analyisis

In accordance with our previous study [81], the determination of Si, macro- and microelements (boron-B, calcium-Ca, iron-Fe, potassium-K, magnesium-Mg, manganese-Mn, phosphorus-P, sulfur-S, and zinc-Zn) was carried out by inductively coupled plasma-optical emission spectrometry (ICP-OES), with both axial and radial viewing using an ICPE-9800 system (Shimadzu Corporation, Kyoto, Japan) equipped with an autosampler (AS-10; Shimadzu, Kyoto, Japan). Exactly 200 mg of previously milled, powdered olive leaf material was weighed in chemically inert polytetrafluoroethylene tubes suitable for microwave digestion. The weighed leaf material was suspended in 6 mL of concentrated nitric acid and 2 mL of 30% hydrogen peroxide per sample and placed in a microwave (Ethos UP, Milestone SRL, high performance microwave digestion system, Bergamo, Italy) for 40 min at 1800 W and 200 °C. Upon completion, the digested contents from the tubes were diluted with 17.5 mL of deionized water (type I) and transferred to the enclosed plastic tubes, reaching a total volume of 25 mL. One replication per digestion method was performed for each sample. The samples were stored at 4 °C until analysis. The method accuracy evaluation was carried out using four certified reference materials from the WEPAL dried plant material program (WEPAL, Wageningen, The Netherlands). The operating parameters were as follows: RF power was set at 1.15 kW, the plasma flow rate was 12 L/min, the auxiliary gas flow rate was 0.5 L/min, and the nebulizer flow rate was 0.5 L/min. Sample solutions were introduced into the plasma using a concentric nebulizer and a cyclonic-type spray chamber. Argon was used to purge the optics and to form the plasma. Nitrogen content was determined according to the Kjeldahl method, as described in a previous study [82].

### 4.12. Statistical Analysis

The experiment was conducted as random block design in five replicates. A three-way analysis of variance (ANOVA) was performed for all analyzed data, with cultivar, treatment, and sampling time as the main factors. Multiple comparisons of means were based on Tukey’s test at *p* ≤ 0.05. Two different types of multivariate analyses were employed. Principal component analysis (PCA) was performed on the correlation matrix, extracting only the principal components (PCs) with eigenvalues > 1.0. Within each of the selected PCs, only the variables with high factor loadings (>0.6) were considered meaningful [83]. Two separate partial least squares discriminant analyses (PLS-DAs), one for each sampling time, were employed to identify patterns and key contributors to short- and long-term group separation. Each PLS-DA was performed using the metabolite concentrations as predictors (X variables) and the cultivar–treatment combinations as response variables (Y variables). For each PLS-DA, an accompanying variable importance in projection (VIP) scores were calculated for the overall model. ANOVA and post hoc comparisons were performed using Statistica v. 13.4 software (Tibco Software Inc., Palo Alto, CA, USA). Multivariate analysis PCA was performed using PAST v. 3.26 software [84], while the two PLS-DA were performed on auto-scaled data using MetaboAnalyst v. 6.0 (University of Alberta, Canada).

## 5. Conclusions

Our findings reveal that silicon’s impact on olive leaf phytochemistry is both genotype-dependent and stress-related. Specifically, while being effective in altering and even improving the phytochemical composition of olive leaves, Si appears to do so in a manner that adheres to each genotype’s metabolic foundation. Moreover, the intensity of environmental constraints and a genotype’s inherent sensitivity to them, may influence silicon’s capacity to mediate significant phytochemical alterations. If the conditions do not sufficiently challenge the metabolic steady state of a given cultivar or trigger its phytochemical adaptive mechanisms, silicon’s impact on its phytochemical composition may appear negligible. In contrast, if a given cultivar’s metabolism is challenged consistently, silicon’s beneficial effects may prove not only immediate but even long-lasting. This can lead to a progressive increase in key protective metabolites like oleuropein. The extent of silicon’s effectiveness seems to be tied to a genotype’s metabolic foundation and its overall versatility and sensitivity, making it inseparable from stress and its intensity. With respect to physiological indices, our findings suggest that Si may be utilized differently among olive cultivars. In addition, certain effects of foliar-applied Si on the phytochemical composition of locally treated leaves can extend to untreated juvenile foliage, with intricate effects on shoot translocation of some essential nutrients.

## Figures and Tables

**Table 1:** Content of silicon (Si) and mineral nutrients in local leaves ^†^ of control and Si-treated olive plantlets (T) from two different cultivars (Cv.), sampled at two different sampling times (ST), 15 and 90 days after the final Si treatment (DAT).

Source of Variation	Si (mg/kg DW)		Macronutrients (g/kg DW)							Micronutrients (mg/kg DW)			
			N	P	K	Ca	Mg	S		B	Fe	Mn	Zn
Cultivar													
Istarska bjelica	136.69 ± 8.47		14.15 ± 0.34 ^b^	0.81 ± 0.02	13.81 ± 0.32	9.54 ± 0.24 ^b^	0.70 ± 0.01 ^b^	0.88 ± 0.01 ^b^		8.43 ± 0.18 ^a^	34.49 ± 20.66	40.43 ± 2.04	9.22 ± 0.26 ^b^
Leccino	137.74 ± 7.42		16.02 ± 0.31 ^a^	0.85 ± 0.02	14.33 ± 0.47	12.84 ± 0.40 ^a^	1.01 ± 0.03 ^a^	1.10 ± 0.02 ^a^		7.26 ± 0.09 ^b^	10.55 ± 1.58	45.12 ± 1.75	14.97 ± 0.33 ^a^
Treatment													
Control	107.55 ± 4.18 ^b^		14.82 ± 0.35	0.81 ± 0.02	14.03 ± 0.42	11.26 ± 0.54	0.86 ± 0.04	1.04 ± 0.03		8.04 ± 0.23 ^a^	32.90 ± 20.71	42.36 ± 1.81	11.99 ± 0.70
Si	166.88 ± 5.73 ^a^		15.35 ± 0.40	0.84 ± 0.01	14.10 ± 0.39	11.11 ± 0.39	0.85 ± 0.03	0.98 ± 0.02		7.65 ± 0.10 ^b^	12.14 ± 1.88	43.20 ± 2.10	12.20 ± 0.62
Sampling time													
15 DAT	141.75 ± 9.84		15.99 ± 0.31 ^a^	0.90 ± 0.01 ^a^	15.80 ± 0.25 ^a^	10.28 ± 0.38 ^b^	0.89 ± 0.04 ^a^	1.04 ± 0.03 ^a^		7.88 ± 0.13	31.16 ± 20.79	41.51 ± 1.93	11.76 ± 0.63
90 DAT	132.67 ± 5.31		14.17 ± 0.35 ^b^	0.76 ± 0.01 ^b^	12.33 ± 0.10 ^b^	12.10 ± 0.48 ^a^	0.82 ± 0.03 ^b^	0.95 ± 0.02 ^b^		7.81 ± 0.22	13.89 ± 1.78	44.04 ± 1.96	12.43 ± 0.69
Cv.	n.s.		***	n.s.	n.s.	***	***	***		***	n.s.	n.s.	***
T	***		n.s.	n.s.	n.s.	n.s.	n.s.	n.s.		*	n.s.	n.s.	n.s.
ST	n.s.		***	***	***	***	*	***		n.s.	n.s.	n.s.	n.s.
Cv. × T	n.s.		n.s.	n.s.	n.s.	n.s.	n.s.	*		**	n.s.	n.s.	n.s.
Cv. × ST	n.s.		n.s.	n.s.	n.s.	n.s.	n.s.	***		n.s.	n.s.	n.s.	n.s.
T × ST	**		n.s.	n.s.	n.s.	n.s.	n.s.	n.s.		n.s.	n.s.	n.s.	n.s.
Cv. × T × ST	n.s.		n.s.	n.s.	n.s.	n.s.	n.s.	n.s.		n.s.	n.s.	n.s.	n.s.

^†^ The term ‘local leaves’ refers to directly treated leaves which were exposed to control and silicon treatments, respectively. Results are expressed as means ± standard errors. Different lowercase letters in a row represent statistically significant differences between mean values for each main effect at *p* ≤ 0.05, obtained by a three-way ANOVA and Tukey’s test. Significance: n.s.—not significant, ***—*p* < 0.001, **—*p* < 0.01, *—*p* < 0.05. DW: dry weight.

**Table 2:** Concentrations of total phenols, simple phenolic alcohols, phenolic acids, terpenoids, secoiridoids, and flavonoids in systemic leaves ^†^ of two olive cultivars—Istarska bjelica and Leccino—from control and Si-treated plantlets, sampled at the end of the experimental period.

**Source of** **variation**	**Total phenolics**	**Simple phenolic alcohols**			**Phenolic acids**				**Terpenoids**
		**Hydroxytyrosol**	**Tyrosol**		**Caffeic**	**Ferulic**	**Verbascoside**		**Oleanolic acid**
	**mg 100 g^−1^ DW**	**mg 100 g^−1^ DW**			**mg 100 g^−1^ DW**				**mg 100 g^−1^ DW**
Cultivar (Cv.)									
Istarska bjelica	3021.55 ± 136.74	15.11 ± 1.17 ^b^	13.29 ± 0.81		5.61 ± 0.31 ^b^	2.80 ± 0.2	12.10 ± 0.90 ^b^		1430.89 ± 61.45 ^a^
Leccino	3409.84 ± 105.01	30.01 ± 2.00 ^a^	14.87 ± 0.61		8.18 ± 0.17 ^a^	2.43 ± 0.14	15.04 ± 2.27 ^a^		1172.68 ± 97.62 ^b^
Treatment (T)									
Control	3086.26 ± 99.31	20.88 ± 3.06	13.41 ± 0.69		6.69 ± 0.49	2.55 ± 0.21	12.17 ± 1.91 ^b^		1320.41 ± 99.47
Si	3345.13 ± 156.57	24.25 ± 2.78	14.75 ± 0.77		7.09 ± 0.48	2.68 ± 0.14	14.98 ± 0.86 ^a^		1283.16 ± 83.89
Cv.	n.s.	***	n.s.		**	n.s.	*		*
T	n.s.	n.s.	n.s.		n.s.	n.s.	*		n.s.
Cv. × T	n.s.	n.s.	n.s.		n.s.	n.s.	**		n.s.
**Source of variation**	**Secoiridoids**		**Flavonoids**						
	**Oleuropein**		**Apigenin**	**Apigenin-O**	**Luteolin**	**Luteolin-O**		**Rutin**	**Diosmetin**
	**mg 100 g^−1^ DW**		**mg 100 g^−1^ DW**						
Cultivar (Cv.)									
Istarska bjelica	1339.05 ± 330.97		1.77 ± 0.18 ^b^	19.83 ± 1.16 ^b^	8.43 ± 0.78 ^b^	559.32 ± 24.34 ^b^		64.54 ± 5.01 ^a^	4.58 ± 0.25 ^b^
Leccino	1421.66 ± 153.54		11.10 ± 1.96 ^a^	77.04 ± 6.9 ^a^	21.25 ± 3.2 ^a^	676.31 ± 14.86 ^a^		51.86 ± 3.58 ^b^	7.22 ± 0.64 ^a^
Treatment (T)									
Control	950.80 ± 73.86 ^b^		8.51 ± 2.63 ^a^	53.29 ± 13.12	18.16 ± 3.89 ^a^	621.86 ± 35.38		63.76 ± 5.06 ^a^	6.14 ± 0.85
Si	1809.91 ± 57.26 ^a^		4.37 ± 0.90 ^b^	43.58 ± 7.29	11.52 ± 1.56 ^b^	613.77 ± 17.83		52.64 ± 3.78 ^b^	4.45 ± 0.35
Cv.	n.s.		***	***	***	**		*	**
T	*		*	n.s.	*	n.s.		*	n.s.
Cv. × T	n.s.		*	*	*	n.s.		n.s.	n.s.

^†^ The term ‘systemic leaves’ refers to juvenile, untreated leaves that developed from plantlets previously exposed to control and silicon treatments, respectively. Shown are means ± standard errors. Different letters indicate significant differences between mean values for each main effect (*p* < 0.05, two-way ANOVA with Tukey’s test). Significance: n.s.—not significant, ***—*p* < 0.001, **—*p* < 0.01, *—*p* < 0.05. DW: dry weight, Apigenin-O: Apigenin-7-O-glucoside, Luteolin-O: Luteolin-7-O-glucoside.

**Table 3:** Content of silicon (Si) and mineral nutrients in systemic leaves ^†^ of two different olive cultivars—Istarska bjelica and Leccino (Cv.)—previously exposed to control and Si treatments (Ts).

Source of Variation	Si (mg/kg DW)		Macronutrients (g/kg DW)							Micronutrients (mg/kg DW)			
			N	P	K	Ca	Mg	S		B	Fe	Mn	Zn
Cultivar (Cv.)													
Istarska bjelica	59.27 ± 5.3		14.22 ± 0.47 ^b^	0.93 ± 0.01 ^b^	13.6 ± 0.63 ^b^	10.02 ± 0.44 ^b^	0.92 ± 0.03 ^b^	1.1 ± 0.02 ^b^		11.67 ± 1.04 ^a^	12.5 ± 4.99	71.7 ± 4.44	13.13 ± 0.56 ^b^
Leccino	58.77 ± 3.29		16.44 ± 0.49 ^a^	1.12 ± 0.02 ^a^	15.57 ± 0.38 ^a^	12.5 ± 0.49 ^a^	1.08 ± 0.03 ^a^	1.23 ± 0.03 ^a^		8.14 ± 0.28 ^b^	5.5 ± 1.4	75.88 ± 2.69	17.53 ± 0.74 ^a^
Treatment (T)													
Control	56.19 ± 5.72		15.32 ± 0.23	1.03 ± 0.03	14.95 ± 0.68	11.67 ± 0.74	1.02 ± 0.04	1.19 ± 0.04		11.53 ± 1.06 ^a^	11.6 ± 4.91	74.19 ± 4.38	15.49 ± 1.06
Si	61.85 ± 2.2		15.34 ± 0.79	1.02 ± 0.03	14.22 ± 0.47	10.85 ± 0.37	0.98 ± 0.03	1.13 ± 0.03		8.28 ± 0.19 ^b^	6.44 ± 1.96	73.39 ± 2.92	15.18 ± 0.86
Cv.	n.s.		***	***	*	**	**	*		***	n.s.	n.s.	**
T	n.s.		n.s.	n.s.	n.s.	n.s.	n.s.	n.s.		***	n.s.	n.s.	n.s.
Cv. × T	n.s.		***	n.s.	n.s.	n.s.	n.s.	n.s.		***	n.s.	n.s.	n.s.

^†^ The term ‘systemic leaves’ refers to juvenile, untreated leaves that developed from plantlets previously exposed to control and silicon treatments, respectively. Shown are means ± standard errors. Different letters indicate significant differences between mean values for each main effect (*p* < 0.05, two-way ANOVA with Tukey’s test). Significance: n.s.—not significant, ***—*p* < 0.001, **—*p* < 0.01, *—*p* < 0.05. DW—dry weight.

**Table 4:** Leaf spectral reflectance indices used for in situ evaluation of each cultivar’s physiological state in response to a given treatment during the experiment.

Abbreviation	Index	Equation	Reference
WBI	Water band index	(R_900_/R_970_)	[71]
PRI	Photochemical reflectance index	(R_531_ − R_570_)/(R_531_ + R_570_)	[72]
PSRI	Plant senescence reflectance index	(R_680_ − R_500_)/R_750_	[73]
CRI 1	Carotenoid reflectance index 1	(1/R_510_) − (1/R_550_)	[74]
CRI 2	Carotenoid reflectance index 2	(1/R_510_) − (1/R_700_)	[74]
CNDVI	Chlorophyll normalized difference vegetation index	(R_750_ − R_705_)/(R_750_ + R_705_)	[75]
NDVI	Normalized difference vegetation index	(R_800_ − R_680_)/(R_800_+R_680_)	[75]
NPQI	Normalized phaeophytinization index	(R_415_ − R_435_)/(R_415_ + R_435_)	[76]
SIPI	Structure intensive pigment index	(R_800_ − R_445_)/(R_800_ − R_680_)	[77]
VREI	Vogelmann red edge index	(R_734_ − R_747_)/(R_715_ + R_720_)	[78]
Ctr	Carter index	(R_695_/R_420_)	[79]
ZMI	Zarco-Tejada & Miller index	(R_750_/R_710_)	[80]

Each ‘R’ value stands for specific wavelength of reflected light indicated in subscript.

## Data Availability

Data contained within the article and the Supplementary Materials.

## Supplementary Materials

The following supporting information can be downloaded at: https://www.mdpi.com/article/10.3390/plants14091282/s1. Table S1. Concentrations of total phenols, simple phenolic alcohols, phenolic acids, and terpenoids in local leaves of two olive cultivars under control and foliar silicon (Si) treatments (T), sampled at 15 and 90 days (ST) after the treatment (DAT). Table S2. Concentrations of secoiridoids, and flavonoids in local leave of two olive cultivars under control and foliar silicon (Si) treatments (T), sampled at 15 and 90 days (ST) after the treatment (DAT). Table S3. Loadings table-principal component analysis (PCA), and partial least squares-discriminant analyses (PLS-DA). Figure S1. Significant two way interactions of: (a) treatment x sampling time on silicon (Si) content, (b) cultivar × treatment on boron (B) content, and (c) cultivar x sampling time for sulfur (S) content in local leaves of two olive cultivars under control (C) and silicon (Si) treatment. Table S4. Spectral reflectance indices of two olive cultivars under control and foliar silicon (Si) treatments (T), measured prior to the first sampling time, 15 days after treatment. Table S5. Spectral reflectance indices of two olive cultivars under control and foliar silicon (Si) treatments (T), measured prior to the second sampling time, 90 days after treatment.
