# Supplementary material for: Silicon-Mediated Modulation of Olive Leaf Phytochemistry: Genotype-Specific and Stress-Dependent Responses
Source: Plants (Basel). 2025 Apr 23;14(9):1282. doi: 10.3390/plants14091282 (PMC12073494; doi:10.3390/plants14091282)
Supplement: Supplementary file 1 [file plants-14-01282-s001.zip › plants-3553878-supplementary.pdf]

Table S1. Concentrations of total phenols, simple phenolic alcohols, phenolic acids, and terpenoids in local leaves† of two olive cultivars (Cv.) (Istarska bjelica and Leccino) under control and foliar silicon (Si) treatments (T), sampled at 15 and 90 days (ST) after the treatment (DAT).

Source of variation	Total phenols	Simple phenolic alcohols		Phenolic acids			Terpenoids
		Hydroxytyrosol	Tyrosol	Caffeic acid	Ferulic acid	Verbascoside	Oleanolic acid
	mg 100 g^{-1} DW	mg 100 g^{-1} DW		mg 100 g^{-1} DW			mg 100 g^{-1} DW
Cultivar (Cv.)							
Istarska bjelica	4617.75 ± 125.89 ^{b}	22.37 ± 1.97 ^{b}	18.32 ± 0.97	20.72 ± 0.71 ^{a}	4.04 ± 0.30 ^{b}	1022.47 ± 74.84 ^{a}	1560.01 ± 89.13 ^{a}
Leccino	5336.81 ± 220.22 ^{a}	37.07 ± 2.93 ^{a}	16.76 ± 0.74	18.64 ± 1.02 ^{b}	5.46 ± 0.15 ^{a}	815.99 ± 106.42 ^{b}	1431.96 ± 71.49 ^{b}
Treatment (T)							
Control	4537.14 ± 130.56 ^{b}	27.74 ± 2.29 ^{b}	18.76 ± 0.97 ^{a}	18.73 ± 0.81	4.89 ± 0.26	770.52 ± 90.29 ^{b}	1485.02 ± 76.64
Si	5417.42 ± 201.28 ^{a}	31.7 ± 3.53 ^{a}	16.32 ± 0.68 ^{b}	20.62 ± 0.95	4.61 ± 0.31	1067.94 ± 87.00 ^{a}	1419.98 ± 115.99
Sampling time (ST)							
15 DAT	4547.40 ± 142.06 ^{b}	39.31 ± 2.48 ^{a}	19.69 ± 0.90 ^{a}	18.87 ± 0.85	5.05 ± 0.27 ^{a}	802.73 ± 98.28 ^{b}	1781.75 ± 50.16 ^{a}
90 DAT	5407.16 ± 195.75 ^{a}	20.13 ± 1.53 ^{b}	15.39 ± 0.50 ^{b}	20.48 ± 0.93	4.45 ± 0.29 ^{b}	1035.72 ± 83.43 ^{a}	1123.25 ± 74.24 ^{b}
Cv.	***	***	n.s.	*	***	n.s.	**
T	***	**	*	n.s.	n.s.	*	n.s.
ST	*	***	***	n.s.	**	*	***
Cv. x T	*	*	n.s.	n.s.	***	n.s.	*
Cv. x ST	*	**	n.s.	n.s.	***	**	n.s.
T x ST	n.s.	***	n.s.	*	***	***	**
Cv. x T x ST	n.s.	**	n.s.	***	***	***	*

†The term 'local leaves' refers to directly treated leaves, exposed to control and silicon treatments, respectively. Shown are means ± standard errors. Different letters in a column represent statistically significant differences between mean values for each main effect at $p < 0.05$ obtained by three-way ANOVA and Tukey's post-hoc test. Significance: n.s.—not significant, ***—$p < 0.001$, **—$p < 0.01$, *—$p < 0.05$. DW—dry weight.

Table S2. Concentrations of secoiridoids, and flavonoids in local leaves[†] of two olive cultivars (Cv.) (Istarska bjelica and Leccino) under control and foliar silicon (Si) treatments (T), sampled at 15 and 90 days (ST) after the treatment (DAT).

Source of variation	Secoiridoids	Flavonoids					
	Oleuropein	Apigenin	Apigenin-O	Luteolin	Luteolin-O	Rutin	Diosmetin
	mg 100 g^{-1} DW	mg 100 g^{-1} DW					
Cultivar (Cv.)							
Istarska bjelica	3612.72 ± 276.16 [b]	1.65 ± 0.107 [b]	34.77 ± 1.28 [b]	9.47 ± 1.16	762.74 ± 22.28 [b]	129.15 ± 5.50 [a]	22.19 ± 0.80 [b]
Leccino	5050.66 ± 533.14 [a]	6.46 ± 0.94 [a]	122.40 ± 7.69 [a]	9.70 ± 1.33	957.02 ± 25.14 [a]	93.78 ± 6.76 [b]	29.16 ± 1.96 [a]
Treatment (T)							
Control	3011.58 ± 302.92 [b]	4.40 ± 1.02 [a]	79.61 ± 12.51	9.84 ± 1.31	884.25 ± 35.95	117.24 ± 7.37	24.27 ± 1.18 [b]
Si	5651.80 ± 373.78 [a]	3.71 ± 0.67 [b]	77.56 ± 10.30	9.33 ± 1.17	835.51 ± 27.69	105.69 ± 7.15	27.08 ± 2.04 [a]
Sampling time (ST)							
15 DAT	3616.66 ± 427.15 [b]	6.06 ± 1.03 [a]	93.13 ± 12.62 [a]	14.05 ± 0.94 [a]	889.56 ± 21.37	128.61 ± 5.21 [a]	20.48 ± 0.51 [b]
90 DAT	5046.73 ± 422.65 [a]	2.05 ± 0.15 [b]	64.04 ± 9.01 [b]	5.13 ± 0.39 [b]	830.21 ± 39.65	94.33 ± 7.08 [b]	30.87 ± 1.63 [a]
Cv.	***	***	***	n.s.	***	***	***
T	***	*	n.s.	n.s.	n.s.	n.s.	***
ST	***	***	***	***	n.s.	***	***
Cv. x T	***	**	n.s.	**	n.s.	n.s.	**
Cv. x ST	***	***	***	n.s.	n.s.	*	***
T x ST	*	*	n.s.	n.s.	n.s.	n.s.	**
Cv. x T x ST	***	*	n.s.	***	*	n.s.	**

[†]The term ‘local leaves’ refers to directly treated leaves, exposed to control and silicon treatments, respectively. Shown are means ± standard errors. Different letters in a column represent statistically significant differences between mean values for each main effect at $p < 0.05$ obtained by three-way ANOVA and Tukey’s post-hoc test. Significance: n.s.—not significant, ***—$p < 0.001$, **—$p < 0.01$, *—$p < 0.05$. DW—dry weight, Apigenin-O – Apigenin-7-O-glucoside, Luteolin-O – Luteolin-7-O-glucoside.

Table S3. Loadings table – principal component analysis (PCA), and partial least squares-discriminant analyses (PLS-DA).

**Statistic**	**PCA loadings***		**PLS-DA 15 DAT loadings**		**PLS-DA 90 DAT loadings**	
	**PC1**	**PC2**	**PLSC1**	**PLSC2**	**PLSC1**	**PLSC2**
Eigenvalue	4.68	3.94	4.79	2.08	5.79	1.85
% variance	32.6	27.5	38.8	17.2	44.51	14.20
Cumulative %	32.6	60.1	38.8	56.00	44.51	58.71
**Factor loading**						
Variables						
Hydroxytyrosol	**0.80**	0.33	**0.40**	0.14	**0.33**	-0.19
Tyrosol	0.43	-0.34	-0.24	**-0.31**	0.008	-0.13
Caffeic acid	-0.44	-0.16	-0.28	-0.12	0.05	**0.60**
Ferulic acid	0.27	0.54	0.10	**-0.52**	**0.38**	0.02
Verbascoside	**-0.61**	-0.08	-0.26	**0.41**	0.08	**0.28**
Oleanolic acid	**0.63**	-0.52	-0.17	**0.31**	-0.22	**-0.39**
Oleuropein	-0.47	**0.61**	0.10	**0.53**	**0.36**	0.19
Apigenin	**0.81**	0.36	**0.38**	-0.12	**0.34**	-0.04
Apigenin-7-O-glucoside	**0.65**	**0.70**	**0.43**	-0.07	**0.33**	-0.21
Luteolin	**0.72**	-0.27	-0.03	0.12	-0.02	0.21
Luteolin-7-O-glucoside	0.51	0.54	**0.40**	-0.24	0.23	**-0.48**
Diosmetin	**-0.60**	**0.70**	0.25	0.05	**0.37**	0.12
Rutin	0.41	**-0.75**	-0.25	-0.02	**-0.34**	-0.02

*PCA loadings are expressed in Pearson's correlation coefficients. The PLS-DA loadings are expressed as correlation-scaled loadings, representing the correlation between the variable and the latent component, scaled by the standard deviation of the variable.

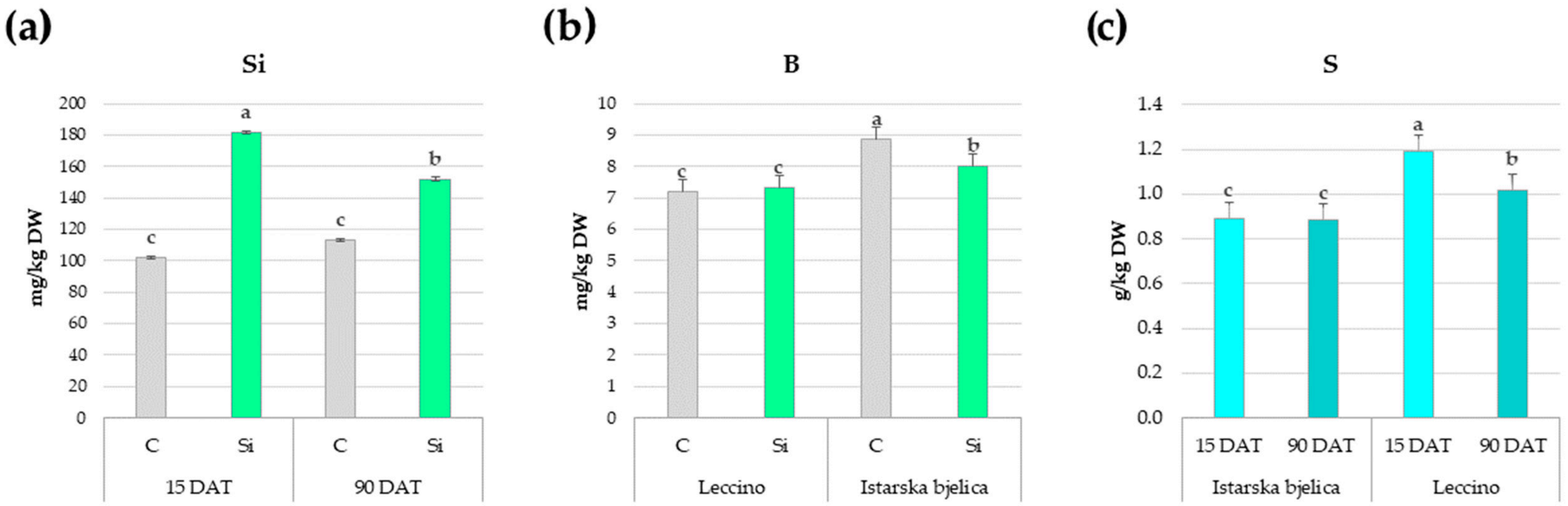

Figure S1. Significant two way interactions of: (**a**) treatment x sampling time on silicon (Si) content, (**b**) cultivar x treatment on boron (B) content, and (**c**)**)** cultivar x sampling time for sulfur (S) content in local leaves of two olive cultivars, Istarska bjelica and Leccino under control (C) and silicon (Si) treatment. Different letters above bars represent statistically significant differences between mean values at $p < 0.05$ obtained by a two-way ANOVA and Tukey's test.

Table S4. Spectral reflectance indices of two olive cultivars (Cv.), Istarska bjelica and Leccino under control and foliar silicon (Si) treatments (T), measured prior to the first sampling time, 15 days after treatment.

Source of variation	WBI	PRI	PSRI	CRI 1	CRI 2	CNDVI	NDVI	NPQI	SIPI	VREI	Ctr	ZMI
**Cultivar (Cv.)**												
Istarska bjelica	0.99 ± 0.01 [b]	0.05 ± 0.005 [a]	-0.03 ± 0.008 [b]	0.06 ± 0.006	0.06 ± 0.15 [b]	0.52 ± 0.009	0.84 ± 0.01	0.07 ± 0.03	0.53 ± 0.05 [b]	1.48 ± 0.01 [a]	0.45 ± 0.06 [b]	2.41 ± 0.04 [b]
Leccino	1.02 ± 0.007 [a]	0.03 ± 0.003 [b]	-0.02 ± 0.004 [a]	0.3 ± 0.15	0.13 ± 0.06 [a]	0.5 ± 0.007	0.83 ± 0.008	0.04 ± 0.01	0.7 ± 0.03 [a]	1.44 ± 0.01 [b]	0.72 ± 0.09 [a]	2.29 ± 0.03 [a]
**Treatment (T)**												
Control	0.95 ± 0.01 [b]	0.03 ± 0.002 [b]	0.002 ± 0.003 [a]	0.33 ± 0.15	0.33 ± 0.14 [a]	0.51 ± 0.006	0.82 ± 0.008	0.014 ± 0.02 [b]	0.84 ± 0.01 [a]	1.46 ± 0.009	0.8 ± 0.1 [a]	2.34 ± 0.03
Si	1.06 ± 0.006 [a]	0.06 ± 0.004 [a]	-0.06 ± 0.006 [b]	0.04 ± 0.006	0.04 ± 0.005 [b]	0.51 ± 0.009	0.82 ± 0.01	0.11 ± 0.02 [a]	0.4 ± 0.04 [b]	1.46 ± 0.015	0.37 ± 0.05 [b]	2.36 ± 0.05
Cv.	**	***	*	n.s.	*	n.s.	n.s.	n.s.	***	*	*	*
T	***	***	***	n.s.	*	n.s.	n.s.	**	***	n.s.	**	n.s.
Cv. x T	***	**	***	n.s.	***	n.s.	n.s.	n.s.	***	n.s.	*	n.s.

Shown are means ± standard errors. Different letters in a column represent statistically significant differences between mean values for each main effect at $p < 0.05$ obtained by two-way ANOVA and Tukey's post-hoc test. Significance: n.s.—not significant, ***—$p < 0.001$, **—$p < 0.01$, *—$p < 0.05$.

Table S5. Spectral reflectance indices of two olive cultivars (Cv.), Istarska bjelica and Leccino under control and foliar silicon (Si) treatments (T), measured prior to the second sampling time, 90 days after treatment.

Source of variation	WBI	PRI	PSRI	CRI 1	CRI 2	CNDVI	NDVI	NPQI	SIPI	VREI	Ctr	ZMI
**Cultivar (Cv.)**												
Istarska bjelica	0.97 ± 0.01	0.08 ± 0.004 [a]	-0.055 ± 0.007	0.047 ± 0.007	0.033± 0.007	0.66 ± 0.007	0.85 ± 0.01	0.101 ± 0.02	0.57 ± 0.04 [b]	1.83 ± 0.01	0.37 ± 0.07 [b]	3.55 ± 0.07
Leccino	1.008 ± 0.01	0.06 ± 0.006 [b]	-0.0004 ± 0.028	0.049 ± 0.019	-0.016 ± 0.07	0.65 ± 0.01	0.86 ± 0.01	0.128 ± 0.02	0.71 ± 0.03 [a]	1.76 ± 0.04	0.65 ± 0.09 [a]	3.41 ± 0.06
**Treatment (T)**												
Control	0.99 ± 0.016	0.07 ± 0.006	-0.012 ± 0.03	0.043 ± 0.019	-0.02 ± 0.07	0.66 ± 0.01	0.87 ± 0.01	0.105 ± 0.02	0.65 ± 0.03	1.8 ± 0.04	0.54 ± 0.09	3.5 ± 0.07
Si	0.98 ± 0.01	0.07 ± 0.004	-0.043 ± 0.007	0.052 ± 0.006	0.04 ± 0.006	0.65 ± 0.008	0.84 ± 0.02	0.123 ± 0.02	0.63 ± 0.04	1.79 ± 0.01	0.47 ± 0.08	3.4 ± 0.06
Cv.	n.s.	*	n.s.	n.s.	n.s.	n.s.	n.s.	n.s.	*	n.s.	*	n.s.
T	n.s.	n.s.	n.s.	n.s.	n.s.	n.s.	n.s.	n.s.	n.s.	n.s.	n.s.	n.s.
Cv. x T	n.s.	n.s.	n.s.	n.s.	n.s.	n.s.	n.s.	n.s.	n.s.	n.s.	n.s.	n.s.

Shown are means ± standard errors. Different letters in a column represent statistically significant differences between mean values for each main effect at $p < 0.05$ obtained by two-way ANOVA and Tukey's post-hoc test. Significance: n.s.—not significant, ***—$p < 0.001$, **—$p < 0.01$, *—$p < 0.05$.
